# *Cyberlindnera jadinii* Yeast as a Protein Source for Weaned Piglets—Impact on Immune Response and Gut Microbiota

**DOI:** 10.3389/fimmu.2020.01924

**Published:** 2020-09-02

**Authors:** Leidy Lagos, Alexander Kashulin Bekkelund, Adrijana Skugor, Ragnhild Ånestad, Caroline P. Åkesson, Charles McL. Press, Margareth Øverland

**Affiliations:** ^1^Department of Animal and Aquacultural Sciences, Faculty of Biosciences, Norwegian University of Life Sciences, Aas, Norway; ^2^Department of Food Safety and Infection Biology, Norwegian University of Life Sciences, Oslo, Norway; ^3^Department of Preclinical Sciences and Pathology, Faculty of Veterinary Medicine, Norwegian University of Life Sciences, Oslo, Norway

**Keywords:** post-weaning pig, novel protein, yeast, health, microbiota

## Abstract

Supplying novel feed ingredients for pig production is crucial to enhance food security and decrease the environmental impact of meat production. Several studies have focused on evaluating the beneficial health effects of yeast in pigs. However, its use as a protein source has been partially addressed. Previously, we have shown that yeast at high inclusion levels maintains growth performance and digestibility, while nutrient digestibility, intestinal villi height and fecal consistency were improved. The present study combined microbiome, short-chain fatty acid, and immune parameter analysis to investigate the effect of high inclusion of yeast in diets for post-weaning piglets. Our results showed that yeast did not have a significant impact on the hematological or biochemical parameters in blood. The different immune cell subpopulations isolated from blood and distal jejunal lymph nodes (DJLN) were analyzed by flow cytometry and showed that yeast diet induced an increased number of the subtype of leukocytes CD45+/CD3–/CD8+, a special type of Natural Killer (NK) cells. Also, a very mild to moderate infiltration of neutrophilic granulocytes and lower IgA level were observed in the colon of yeast fed piglets. The microbiome profiling in different compartments of the gastrointestinal tract of piglets was performed using 16S rRNA metabarcoding. The results showed that 40% replacement of dietary protein had a statistically significant effect on the microbial communities in cecum and colon, while the microbial population in ileum and jejunum were not affected. Analysis of predicted microbial metabolic pathways analysis revealed significant upregulation of short-chain fatty acids, ether lipid metabolisms, secondary bile acids, and several other important biosynthesis pathways in cecum and colon of pigs fed yeast. In conclusion, the results showed that diet containing 40% of yeast protein positively shaped microbial community in the large intestine and increased the number of a specific subpopulation of NK cells in the DJLN. These results showed that yeast modulates the microbiome and decreases the secretion of IgA in the colon of post-weaning pigs.

## Introduction

Weaning is a critical period in a pig‘s life due to abrupt dietary, social, and environmental changes (1). This stressful period induces a decreased feed intake resulting in intestinal inflammation, increased susceptibility to infection, unbalanced gut microbiota, and hence post-weaning diarrhea (2). Antibiotic growth promoters (AGPs) have been extensively incorporated into pig feeds to help overcome post-weaning stress; however, cross-resistance of bacteria to antibiotics and the European ban of AGPs (EC 1831/2003) has led to a search for alternatives (3). Given the major role of antibiotics in gut microbiota dysbiosis, awareness in public health, and concerns about the spread of multiresistant bacteria, there is an urgent need to develop non-antibiotic based strategies to restore microbial balance and control diseases associated with the weaning period (4). Accordingly, probiotics and prebiotics have been used as gut microbiota modulators reducing pathogenic colonization, consequently improving animal health and welfare (5).

Supplying novel feed ingredients for pig production is crucial to enhance food security and decrease the environmental impact of meat production. Animal production can no longer continue to depend on protein ingredients that are questioned for both ethical and economic reasons (6). Thus, yeast produced from non-food resources such as second-generation sugars represents a high-quality protein and, as such, is a competitive and sustainable novel feed ingredient (7). Yeast or yeast products have been shown to promote growth performance, modulating gut microbiome and positively affect the immune system, thus reducing post-weaning diarrhea (8). Several studies have focused on evaluating the beneficial health effects of yeast in pigs (9, 10). However, its use has been limited to additives, while its health effects when used as a protein source as been partially addressed. The presence of a non-digestible cell wall in the yeast could have adverse effects on the nutritional value when used at high inclusion levels. Also, the composition and functional properties of different yeast strains can vary substantially depending on the yeast strain and processing conditions (7). Cruz et al. (11) have shown that locally produced dry yeast strain of *Candida utilis* (*C. utilis*), which is the anamorphic name of *Cyberlindnera jadinii*, can replace up to 40% of dietary crude protein while supporting high growth performance, improve digestive function, and thus reducing the incidence of diarrhea.

Gut microbiota plays a crucial role in intestinal morphology, regulation of immunity, digestion of carbohydrates, and health in livestock (12). Moreover, the composition of gut microbiota is affected by many factors, such as age, health status, and diet. For instance, Kiros et al. (13) have shown that the effect of live yeast (*Saccharomyces cerevisiae*) supplementation on the composition of the large intestinal microbiota was more pronounced when provided during the post-weaning period. The results showed that the microbiota composition was phylogenetically more homogeneous than those of control piglets, suggesting that the positive effects attributed to yeast may be partially due to its ability to modify the composition of gut microbiota (14).

The impact of yeast-derived components such as cell wall β-glucans and mannan-oligosaccharides on the gut microbiota in pigs has also been studied. Fouhse and co-workers reported a high relative abundance of *Mitsuokella* and a low relative abundance of *Coprococcus* and *Roseburia* in the cecum of piglets supplemented with yeast-derived mannan-rich fraction (14). While supplementation of yeast ingredients does seem to promote distinct intestinal bacterial groups, the modulation of short-chain fatty acid (SCFA) producing bacteria may be another intrinsic feature of such diets. Acetic, propionic, and butyric acid are among the most abundant SCFAs found in the gut. Acetate production is widely distributed among bacteria, and it is difficult to relate it to a specific bacterial group (15). However, propionic acid is mainly produced by *Prevotellaceae* and *Bacteroidiaceae* families or the *Negativicutes* class. It has been shown that SCFA have several key functions related to gut homeostasis and health (16–18).

The main objective of the present study was to evaluate the effect of inactivated *Cyberlindnera jadinii* yeast when used in higher inclusion levels as a protein source on the microbial profile and metabolites, and immune parameters in post-weaned piglets.

## Results

### Effect of Yeast on Blood Parameter

**Figure 1:**
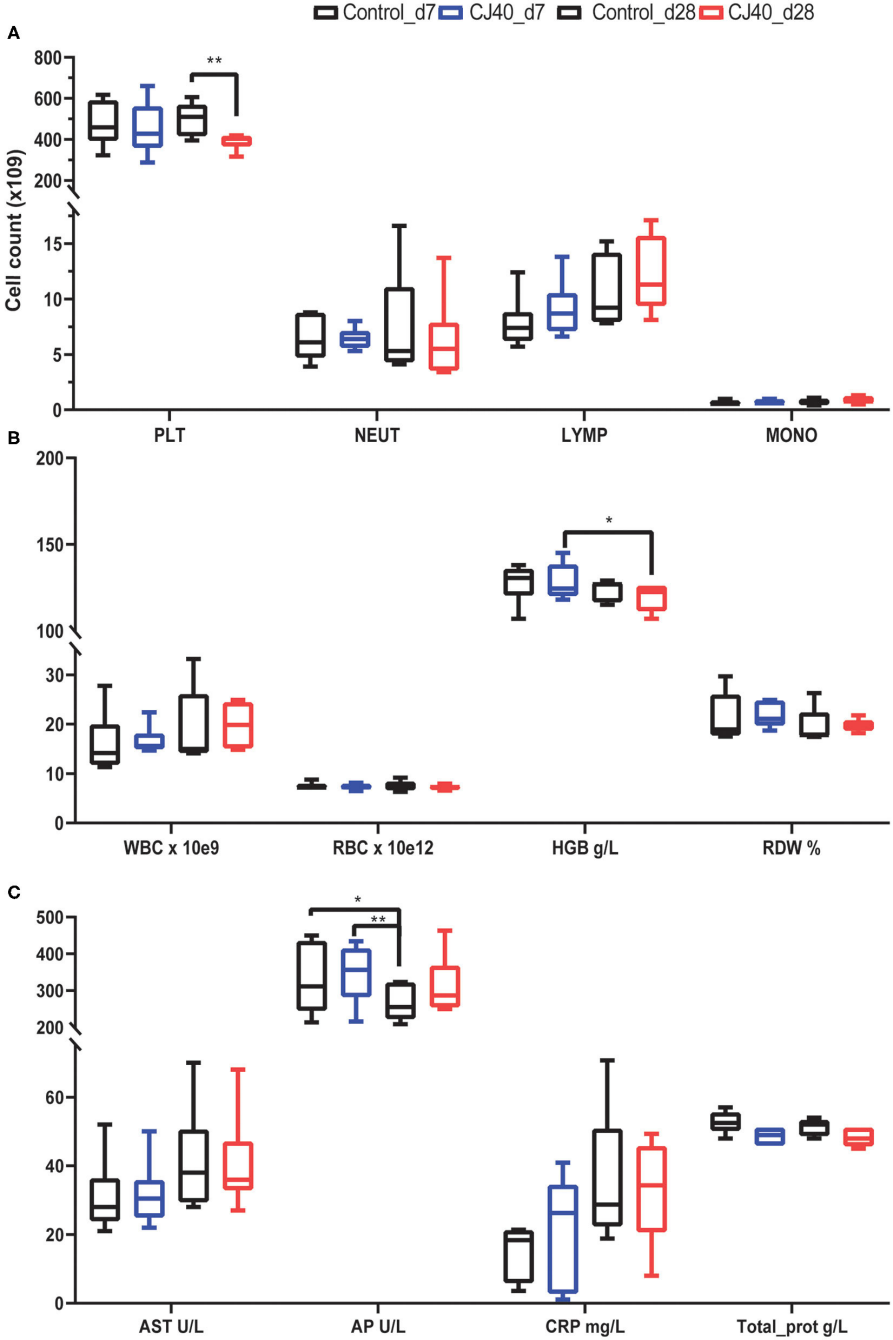
Effect of yeast on selected hematological and biochemical parameters in the blood at 7 and 28 days post-weaning. **(A)** Bar plots showing the number of platelets (PLT), neutrophils (NEUT), lymphocytes (LYMP), and monocytes (MONO) at 7 (d7) or 28 days (28 d) after receiving a control diet or 40% yeast (CJ40). **(B)** Bar plots showing the number of white blood cells (WBC), red blood cells (RBC), the concentration of hemoglobin in grams per Liter (HGB), and percentage of red blood cell distribution width (RDW). **(C)** Biochemical metabolic profile in serum, aspartate aminotransferase (AST), alkaline phosphatase (AP), C-reactive protein (CRP), total protein (Total_prot). *n* = 6 per group. Asterisk represents statistical difference, *p = 0.03, **p = 0.002.

The hematological analysis did not reveal a significant difference in the number of lymphocytes, monocytes, or neutrophils between pigs fed control and experimental diet (CJ40), both at 7 and 28 days post-weaning; however, we observed a significantly lower number of platelets at day 28 on pigs fed yeast (Figure 1A). We also observed that piglets fed yeast showed a substantially lower amount of hemoglobin after 28 days compared to that observed at 7 days (Figure 1B). All parameters are shown in Table S1.

Regarding the biochemical parameters in serum, there were no significant differences in the levels of aspartate aminotransferase (AST); however, the concentration of alkaline phosphatase (AP) decreased in the control pigs at 28 days compared to the control and CJ40 fed pigs at 7 days. In the yeast fed pigs the decrease in AP at 28 days compared to day 7 was no significant (Figure 1C). The results of all biochemical parameters are shown in Table S2.

### Effect of Yeast on Immune Cells

**Figure 2:**
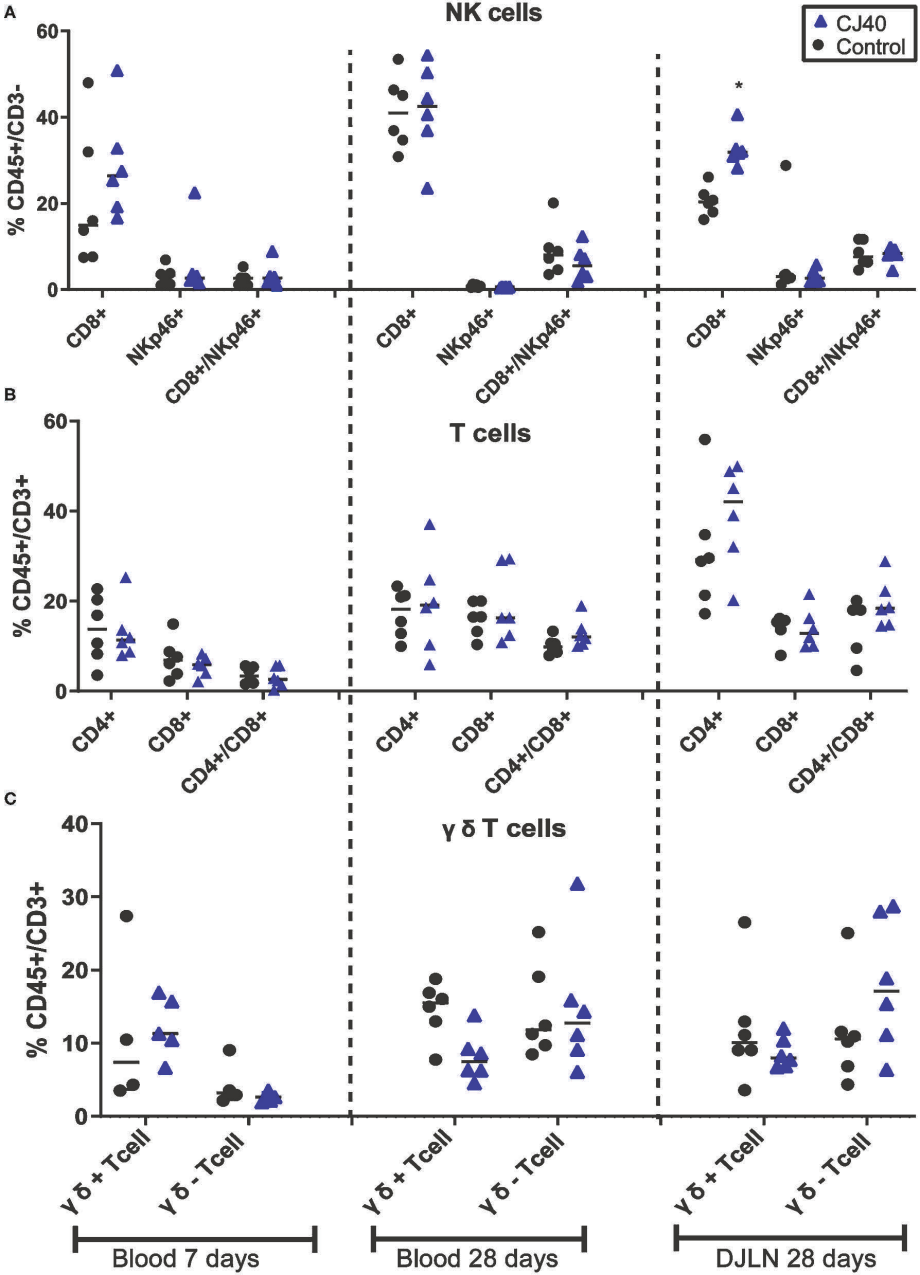
Effect of yeast on relative immune population size in blood and distal jejunal lymph node (DJLN). Flow cytometric analysis of Natural Killers cells **(A)**, T cells **(B)**, and γδ T cells **(C)** out of the total peripheral blood mononuclear cells at 7 and 28 days or DJLN at 28 days. *n* = 6 per group. The asterisk represents the statistical difference, *p = 0.03.

Leukocytes were isolated from blood at 7 and 28 days, distal jejunal lymph node (DJLN) at 28 days and analyzed by flow cytometry. As shown in Figure 2A, the diet containing yeast induced an increasing number of CD3–/CD8+ cells in DJLN at 28 days post-weaning, but this increase was not observed in the blood or other subpopulations of leukocytes (Figures 2B,C). The gating strategy used in this study is presented in Figure S1. There was no statistical difference in plasma levels of IL-1A, IL1B, IL-1RA, IL-4, IL-6, IL-8, IL-10, IL-12, IL-18, and TNFα between the control and experimental group on day 7 (Table 1) and 28 (Table 2) in this experiment, while IL-2 and GMCSF were undetectable at both time points.

**Table 1:** Inflammatory responses of weaned piglets fed a control diet or a 40% yeast protein diet at 7 days (Mean ± SD).

**ng/ml**	**Control**	**CJ40**	***P***
IFNγ	7.5 ± 0.5	8.3 ± 0.8	0.93
IL1a	0.01^a^	0.01^a^	>0.99
IL1b	UD	UD	
IL1ra	0.98 ± 0.3	0.14 ± 0.03	0.99
IL4	UD	UD	
IL6	UD	UD	
IL8	2.22 ± 1.2	0.50 ± 0.02	0.87
IL10	0.06 ± 0.003	0.03 ± 0.002	>0.99
IL12	0.72 ± 0.09	0.87 ± 0.07	>0.99
IL18	0.51 ± 0.19	0.22 ± 0.01	>0.99
TNFα	0.35 ± 0.2	0.35 ± 0.2	>0.99

*The letters indicate that cytokine was detected in just one sample. UD, undetected*.

**Table 2:** Inflammatory responses of weaned piglets fed a control diet or a 40% yeast protein diet at 28 days (Mean ± SD).

**ng/ml**	**Control**	**CJ40**	***P***
IFNγ	6.83 ± 0.8	6.4 ± 0.7	0.99
IL1a	0.01^a^	0.102 ± 0.06	>0.99
IL1b	UD	1.28 ± 0.8	0.77
IL1ra	0.14 ± 0.04	0.8 ± 0.55	0.99
IL4	UD	0.35 ± 0.15	>0.99
IL6	0.07^a^	0.99 ± 0.7	0.99
IL8	1.32 ± 0.53	0.89 ± 0.21	0.99
IL10	0.04 ± 0 003	1.3 ± 0.91	0.73
IL12	0.85 ± 0.08	0.86 ± 0.11	>0.99
IL18	0.34 ± 0.09	1.17 ± 0.73	0.93
TNFα	0.24 ± 0.13	0.11 ± 0.02	>0.99

*The letters indicating that cytokine was detected in just one sample. UD, undetected*.

### Effect of Yeast on the Expression of Genes

The gene expression of CLDN4 and TJP1 with roles in the regulation of tight junctions proteins across the intestinal epithelium was measured in the colon at 28 days post-weaning. In addition, colonic mRNA expression of toll-like receptor, TLR4, was assessed as several previous studies in pigs and poultry reported that dietary yeast supplementation could modulate the expression of pattern recognition receptors in the gut including the TLR4, thus stimulating the innate immune response (19–21). Similarly, the expression of IL1b and IL6 genes encoding the cytokines of the innate immune system was measured to study the effect of yeast on the inflammatory response. However, none of the genes included in the study showed significantly different expression levels between the diets (Figure S2). Because of the observed increase in the NK cell number in the DJLN of pigs fed the yeast diet, the expression of a larger set of genes with the role in immunity and NK cell function was measured. Among the selected genes were those coding for cytokines IL10, IL12a, IL18, IL6, and IL13, receptors of activated NK-cells KLRK1 (NKG2D) and NCR1 (NKp46), chemokines CXCL8 and CCL22 and FOXP3 marker of T reg. Similarly to the qPCR results in the colon, the measured mRNA gene expression profiles in DJLN showed no significant difference between the diets (Figure S3).

### Histology of the Colon

**Figure 3:**
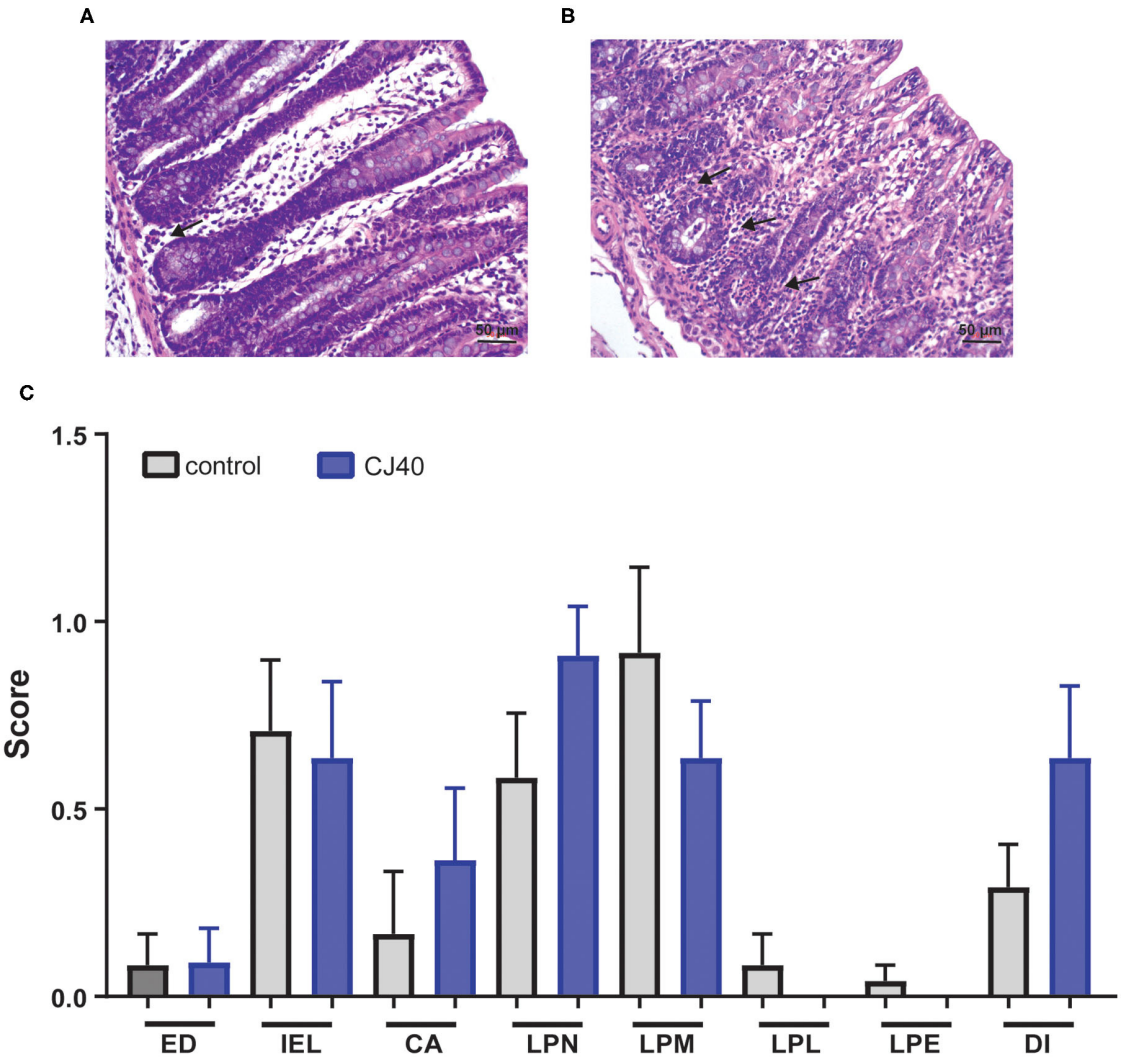
Histology of the colon. **(A)** Colon of piglet fed the control diet, few neutrophils (black arrow) are present in the lamina propria. Bar 50 μm. **(B)** Colon of piglet fed CJ40 yeast diet. A moderate diffuse presence of neutrophils (black arrows) is evident. Bar 50 μm. **(C)** Histopathologic evaluation of colon of piglets fed the control (control) or CJ40 yeast diet. The mean and SD of semi-quantitative scoring of histopathological parameters are presented. No pathology was scored 0, very mild changes were scored 0.5; mild changes 1.0; mild-moderate changes 1.5; and moderate changes 2.0. The histopathological parameters evaluated were epithelial damage (ED); intra-epithelial lymphocytes (IEL); crypt abscess (CA); lamina propria neutrophils (LPN); lamina propria macrophages active (LPM); lamina propria lymphocytes/plasma cells (LPL); lamina propria eosinophils (LPE); and diagnosis colitis (DI). *n* = 12 for the control group and *n* = 11 for yeast fed group. Mann–Whitney testing of parameters did not reveal statistical differences, *p* > 0.05.

Histopathological examination of the colon of piglets fed control or yeast diets revealed only very mild to moderate changes in the intestinal tissues of both groups (Figure 3). Semi-quantitative assessment of changes in the colon showed an increased presence of neutrophils in the lamina propria of piglets fed the yeast diet. A diagnosis of very mild to moderate neutrophilic colitis was made in more piglets from the yeast (8/11) than the control fed group (5/12) (Figure 3). No statistical differences were detected between the mean scores of the histopathological parameters.

### SCFA and IgA

**Figure 4:**
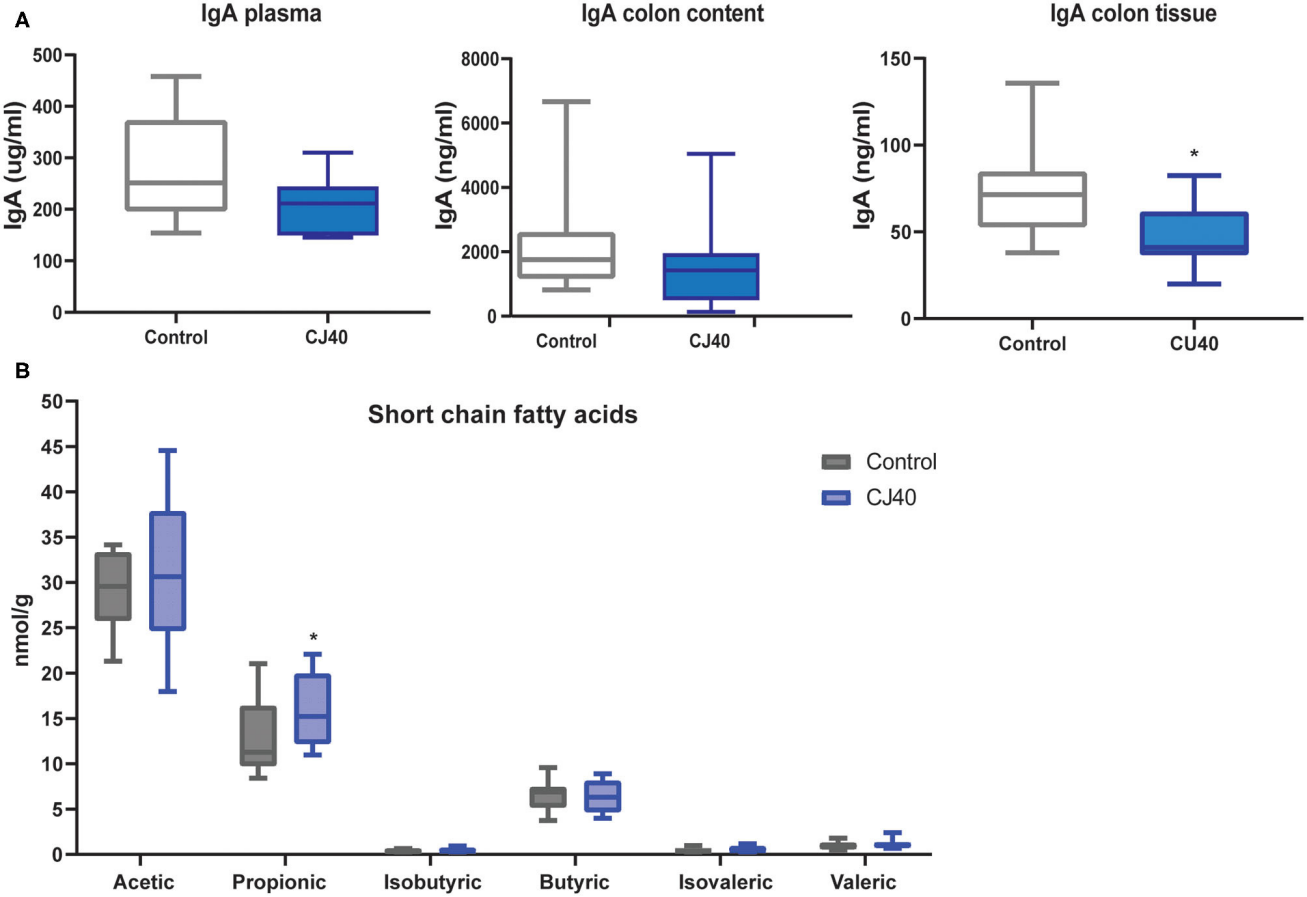
Effect on IgA and short-chain fatty acids in piglets fed 40% yeast or control diet. **(A)** Plasma, colon content, and colon tissue immunoglobulin (IgA) concentration in pigs fed diets supplemented with 40% yeast (CJ40) or the control diet (control) at 28 days after weaning. **(B)** Short-chain fatty acid concentration in the colon digesta of piglets fed 40% yeast (CJ40) or the control diet (control) at 28 days post-weaning. *n* = 12 per group. The asterisk represents significant differences *p* = 0.05.

The short-chain fatty acids (SCFAs) and IgA levels in plasma, colon content and colon tissue were measured by LC-MS and ELISA, respectively. Regarding IgA levels, there was a tendency of lower levels of IgA in plasma (*p* < 0.9) and colon content (*p* < 0.2) in piglets fed yeast compared to the control group (Figure 4A), although the difference was not significant. However, in colon tissue, the levels of IgA were significantly lower (*p* < 0.01) in piglets fed yeast when compared to the piglets fed the control diet (Figure 4A). Moreover, pigs fed yeast showed higher levels of propionic acid in the colon content (*p* < 0.05; Figure 4B). We did not observe a significant difference in the levels of acetic, isobutyric, butyric, isovaleric, or valeric acid.

### Effect of Yeast on Intestinal Microbiota

**Figure 5:**
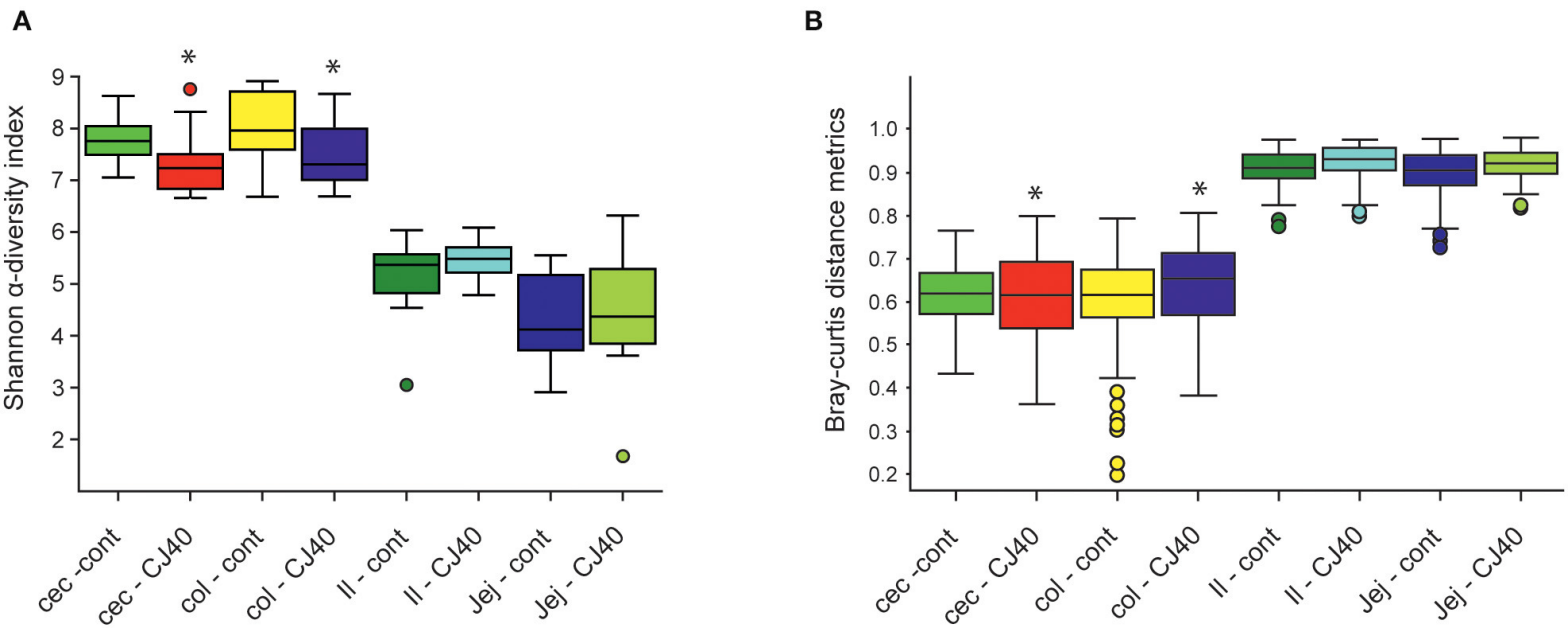
Effects of the diet containing 40% yeast protein on **(A)** α-diversity, **(B)** β-beta diversity of microbial populations in ileum, jejunum, cecum, and colon samples. The statistically significant differences were observed for α-diversity metrics for cecum (Kruskal–Wallis: *H* = 4.32, *p* = 0.04, and *q* = 0.06) and colon (Kruskal–Wallis: *H* = 3.85, *p* = 0.05, *q* = 0.07) samples; β-beta diversity metrics for cecum (PERMANOVA: pseudo—*F* = 2.1, *p* = 0.03, and *q* = 0.04) and colon (PERMANOVA: pseudo—*F* = 1.9, *p* = 0.05, and *q* = 0.06) samples. Asterisk represent significant difference. Inclusion of 40% yeast protein had no statistically significant impact on α-diversity metrics neither for ileum (Kruskal–Wallis: *H* = 0.32, *p* = 0.57, and *q* = 0.59) and jejunum (Kruskal–Wallis: *H* = 0.39, *p* = 0.53, and *q* = 0.58) samples nor for β-beta diversity metrics for ileum (PERMANOVA: pseudo—*F* = 0.52, *p* = 0.901, and *q* = 0.90) and jejunum (PERMANOVA: pseudo—*F* = 0.52, *p* = 0.834, and *q* = 0.86) samples. *n* = 12 per group.

**Figure 6:**
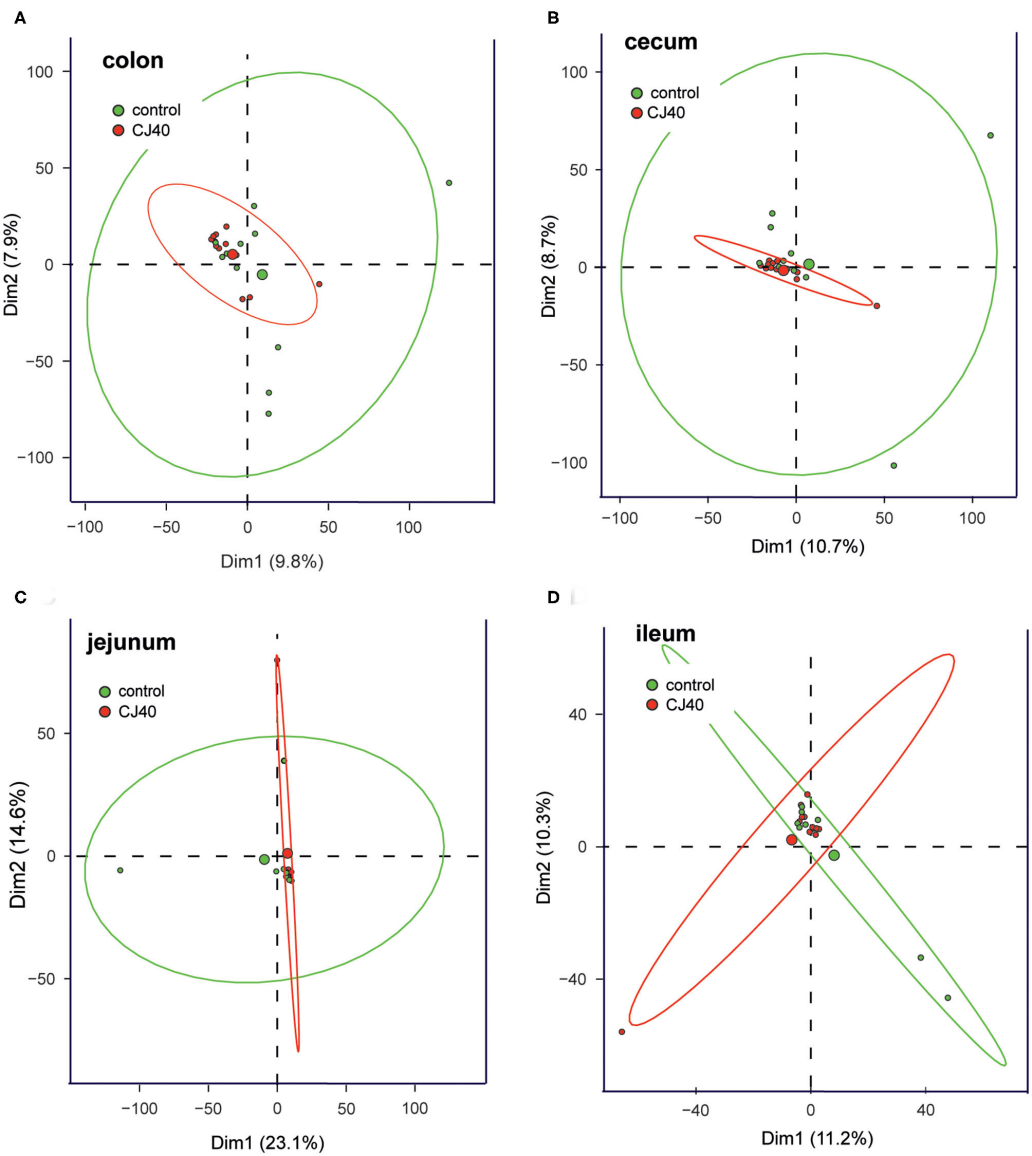
PCA of the effects of the diet containing 40% yeast protein on the relative abundances of microbial communities in **(A)** cecum, **(B)** ileum, **(C)** jejunum, and **(D)** colon samples. The ellipsoids represent confidence intervals (*p* ≥ 95%) and the centroids represent medians for the respective groups of samples. *n* = 12 per group.

Analysis of microbial composition was performed on ileum, jejunum, cecum, and colon contents collected on day 28 of the experiment. The addition of 40% yeast did not significantly affect Shannon α-diversity indexes in ileum and jejunum compared to the control. The α-diversity was slightly but significantly decreased in cecum and colon samples (α = 95%) of the yeast fed piglets (Figure 5A). The Bray–Curtis dissimilarity measurements show that the inclusion of 40% of yeast protein to the diet had no impact on β-diversity in the ileum and jejunum (Figure 5B). Statistically significant differences were observed for the microbial populations in the cecum (PERMANOVA: pseudo—*F* = 2.1, *p* = 0.03, and *q* = 0.04) and colon (PERMANOVA: pseudo—*F* = 1.9, *p* = 0.05, and *q* = 0.06). The PCA analysis shows close clustering of the samples within two-dimensional coordinates (Figures 6A,B). However, no significant difference was observed in jejunum or ileum (Figures 6C,D). We have identified 21 differentially abundant genera in the cecum (Table 3), on average, representing 0.004 and 0.011% of the total OTUs (control and CJ40 diets, respectively). Out of those, 19 taxa retain the same differential abundance profiles in colon samples. *Phocea* spp. and *Roseburia* spp. were not found in colon digesta samples.

**Table 3:** Taxonomic characteristics and relative frequencies of differentially abundant groups of microorganisms identified in cecum digesta samples (AM, arithmetic mean).

	**Control diet (*****n*** **=** **12)**		**CJ40 diet (*****n*** **=** **12)**			
**Genus**	**AM × 10^**−5**^**	**SD × 10^**−5**^**	**AM × 10^**−5**^**	**SD × 10^**−5**^**	**Fold change**	**Gram's**
*Anaerofustis*	0.14	0.25	0.40	0.49	2.91	+
*Bacteroides*	1.33	4.16	1.51	3.54	1.14	–
*Barnesiella*	1.18	3.07	2.52	8.74	2.14	–
*Blautia*	0.11	0.37	1.27	2.39	12.03	+
*Breznakia*	4.52	8.52	11.83	18.65	2.61	+
*Butyricicoccus*	0.44	0.73	0.39	1.25	0.88	+
*Clostridium*	0.64	0.93	3.11	3.53	4.90	+
*Cupriavidus*	42.53	75.17	207.24	181.46	4.87	–
*Dialister*	225.16	432.59	799.12	758.40	3.55	–
*Ethanoligenens*	1.77	4.16	2.65	5.71	1.50	+
*Eubacterium*	0.56	1.29	0.77	1.52	1.37	+
*Fournierella*	0.00	0.00	0.70	1.28	0.70	+
*Mitsuokella*	26.22	29.59	31.36	33.06	1.20	–
*Moraxella*	0.54	1.32	0.72	1.90	1.34	–
*Oscillibacter*	2.10	2.40	1.81	0.90	0.86	–
*Papillibacter*	0.21	0.72	0.26	0.62	1.25	+
*Phascolarctobacterium*	2.44	4.43	3.43	5.31	1.41	–
*Phocea*	0.24	0.82	0.36	0.85	1.54	–
*Prevotella*	43.06	29.59	65.52	30.07	1.52	–
*Roseburia*	0.67	1.62	0.36	1.25	0.54	+
*Streptomyces*	2.11	4.42	0.56	1.95	0.27	+

**Figure 7:**
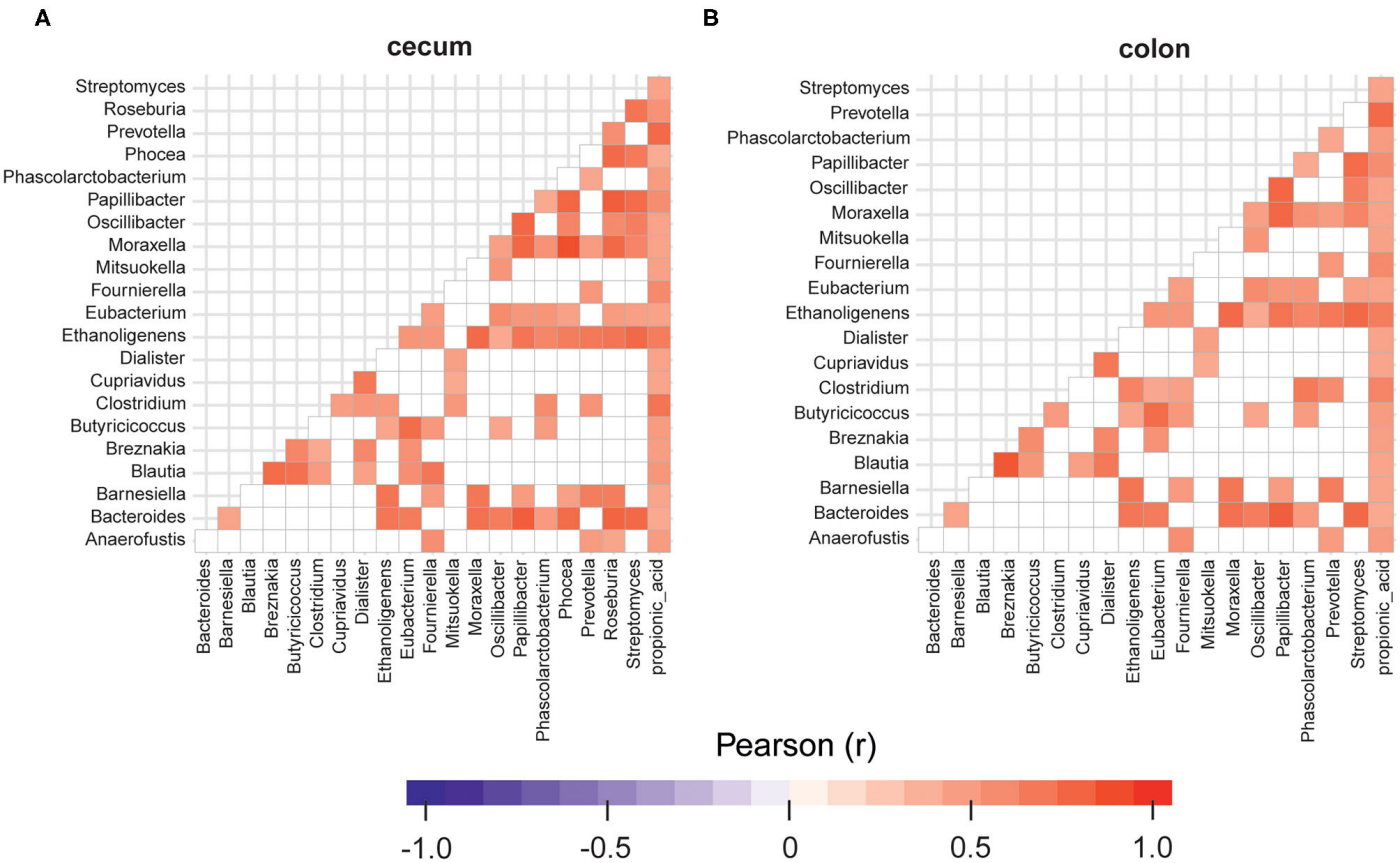
Relationships between the groups of differentially abundant microorganisms identified in cecum **(A)** and colon digesta **(B)** of the animals fed with the diet containing 40% yeast protein. The red color corresponds to a statistically significant positive correlation (*p* ≥ 95%) and the blue color corresponds to a negative correlation. Insignificant values are colored in white. *n* = 12 per group.

The replacement of 40% of protein diet with yeast decreased relative frequencies of *Butyricicoccus, Fournierella, Oscillibacter, Roseburia*, and *Streptomyces* spp., which are described as producers of natural antibiotics and butyrate in the cecum. At the same time, the propionate producing *Phascolarctobacterium* spp. was 1.4 fold more abundant in the cecum samples obtained from the animals fed with CJ40, which correlates well with the increased levels of propionic acid measured in the colon samples, as shown in Figure 7B. We also identify a strong correlation between *Prevotella* and propionic acid in both compartments, colon and cecum (Figures 7A,B). The least noticeable positive fold change in abundance (1.14) was observed for *Bacteroides* spp. The highest positive fold change (12.03) was observed for *Blautia* spp., which possess powerful quorum sensing autoinducers. Analysis of associations between the groups of differentially abundant species revealed predominantly mutualistic relations, and no statistically significant signs of biological competition were discovered in the cecum (Figure 7A) and colon digesta (Figure 7B).

The functional predictions for differentially abundant microbial communities using KEGG database integrated into PICRUSt pipeline revealed a statistically significant (*p* ≤ 0.05) impact of the high yeast protein diets on 23 KEGG bacterial metabolic pathways in the cecum and 121 KEGG in bacterial metabolic pathways in colon. Among those, at least 9 pathways in the cecum (Table 4) and 10 pathways in the colon (Table 5) may directly affect host performance during feeding experiments. The yeast diet had a strong positive effect on the abundance of microbial glycosphingolipid biosynthesis pathways in the cecum (708.42 fold change). In comparison, other pathways were less abundant (0.0004–0.87 fold change). The yeast diets had the greatest impact on the abundances of cyanoamino acid metabolic pathways (3325.98 fold change) and ether lipid metabolic pathways (1,115.18 fold change) in the colon. Moreover, yeast diet had a positive impact on the abundances of fatty acid biosynthesis pathways (6.08–8.69 fold change) and flavonoid biosynthesis pathways (1.89–3.18 fold change). Folate biosynthesis pathways, arachidonic acid biosynthesis pathway, as well as pathways for various glycerolipids and glycerophospholipids, were slightly less abundant in the samples collected from colon of animals fed yeast diet.

**Table 4:** Effect of the 40% yeast protein diet on the PICRUSt predicted frequencies of the selected bacterial metabolic pathways in Cecum (AM, arithmetic mean).

	**Control diet**		**CJ40**			
**Function**	**AM**	**SD**	**AM**	**SD**	**Fold-change**	***p*-value**
Bios. of secondary bile acids	271	140	174,034	45,487	708.42	0.037
Bios. of siderophores	50,780	19,928	165,660	48,498	1.05	0.051
Bios. of unsaturated fatty acids	241,302	51,298	107,624	32,681	0.87	0.003
Bios. of vancomycin group antibiotics	180,378	43,546	78,936	18,449	0.32	0.053
Biotin metabolism	378,470	108,202	73,471	20,620	0.08	0.081
Butanoate metabolism	1,181,758	282,339	67,934	17,397	0.06	0.062
Butirosin and neomycin biosynthesis	185,896	57,132	3,882	2,135	0.01	0.003
C5-Branched dibasic acid metabolism	613,123	165,638	372	444	0.0004	0.009
Carbohydrate digestion and absorption	72,409	24,135	1,126	869	0.0004	0.031

**Table 5:** Effect of 40% yeast protein diet on the PICRUSt predicted frequencies of the selected bacterial metabolic pathways in Colon (AM, arithmetic mean).

	**Control diet**		**CJ40**			
**Function**	**AM**	**SD**	**AM**	**SD**	**Fold-change**	***p*-value**
Cyanoamino acid metabolism	794,450.3	198,432.3	318,739.7	57,151	3,325.98	0.037
Ether lipid metabolism	2,085.25	1,109.904	2,325,427	401,621.4	1,115.18	0.04
Fatty acid biosynthesis	864,818.6	175,356.1	1,449,150	230,273.7	8.69	0.025
Fatty acid metabolism	354,265.4	68,911.81	572,066.5	108,274.5	6.08	0.009
Flavone and flavonol biosynthesis	23,155	11,894.66	2,748,178	488,339.2	3.18	0.019
Flavonoid biosynthesis	3,020.25	2,088.753	79,611.42	21,956.94	1.89	0.005
Folate biosynthesis	945,813.3	204,335.8	334,858.1	52,752.45	0.47	0.049
Glycan biosynthesis and metabolism	73,331.67	18,027.98	194,750.3	61,105.2	0.17	0.008
Glycerolipid metabolism	714,030	149,426.9	97,386.25	20,604.07	0.10	0.003
Glycerophospholipid metabolism	1,160,996	246,872	276.4167	285.2614	0.0038	0.034

## Discussion

In the present study, we investigated whether yeast, when used as an alternative protein source, influence immune function and modulate microbiota in post-weaning pigs. As shown in our previous study (11), high inclusion levels of *Cyberlindnera jadinii* yeast do not affect growth performance or feed intake but increased apparent total tract digestibility of crude protein. In addition, yeast increases intestinal villi height, suggesting an increase in intestinal absorption and gut health. In this study, the effect of high inclusion of yeast on serum and DJLN, gut microbiota composition in four intestinal segments and SCFAs in the colon was investigated in post-weaning piglets. The results showed that yeast regulated the microbiota composition, selectively changing the propionic acid profile in the large intestine and increasing the number of a specific subpopulation of NK cells in the DJLN.

The proper functioning of the immune system is crucial for the transition from milk to solid feed in the weaning period. Regarding the type of immune cells assessed by flow cytometry, yeast diet induced an increased number of the subtype of leukocytes CD45+/CD3–/CD8+ in the DJLN. These cells have been described as NK cells and are numerous in blood and among splenocytes. In lambs, the recruitment of activated NK cells in the intestine is associated with the mobilization of innate immune responses to experimental cryptosporidiosis (22). NK cells participate in the early protective response against pathogens and secrete inflammatory cytokines to activate the adaptive immune system. Porcine NK cells have been shown to possess properties associated with antigen-presenting cells allowing them to stimulate T cell proliferation (23). Liu et al. (24) have shown the impact of early feeding on the number of this type of NK. In the current study, pigs that were fed a diet containing yeast exhibited a higher number of NK cell populations than pig fed the control diet. Because of these results, we assessed the presence of inflammatory cytokines that might be secreted by this subpopulation of cells. Although, we did not observe a significant difference in the secretion of cytokines a numerical increase was observed in the secretion of IL-10 and IL-18 at 28 days in the blood of pigs fed yeast, while a numerical lower secretion of IL-8 at 7 and 28 days was observed in the blood of pigs from the same group. Interleukin 18 is a pro-inflammatory cytokine and is key to promoting the production of interferon-gamma by NK cells against viral, fungal, bacterial and parasitic infections (25). On the other hand, IL-10, which is described as a cytokine synthesis inhibitory factor (26), is an important factor in shaping tolerant NK cells (27).

Regarding IL-8, several studies have reported that IL-8 can enhance the invasion and metastasis of solid tumors (28), and modulate the function of NK cells through the inhibition of receptors such as NKp30, NKG2D, and granzyme B (29). However, it is important to note that we did not measure cytokines in the DJLN, which might explain the low levels found in plasma and suggest a local effect rather than a systemic effect of yeast. Interestingly, a very mild to moderate infiltration of neutrophilic granulocytes was observed in the colon of more of the yeast fed piglets than the piglets fed the control diet. The presence of neutrophils in the lamina propria of the colon could suggest a local pro-inflammatory environment facilitating the mobilization of innate mucosal immune responses during weaning stress (30). Neutrophils were also present in the lamina propria of some piglets in the control group, justifying a diagnosis of very mild to mild colitis. The digestive tract of weaned piglets can be sensitive to antinutritional factors present in ingredients of the control and yeast diets such as soybean meal (31).

The present study indicates that yeast did not have a significant impact on the hematological or biochemical parameters in blood, except for the platelets count. The diet containing a high level of yeast induced significantly lower blood platelet counts at 28 days post-weaning compared with the control group; however, this value was within the value range for a healthy pig (32). Furthermore, the high inclusion level of yeast in the diet did not affect the serum concentration of AST or AP. These two enzymes are known to be increased in response to acute liver injury or liver toxicity (33). Therefore, we suggest that feed yeast for 4 weeks did not induce evident damage to the liver of piglets post-weaning.

Even though immunoglobulin G (IgG) is the main isotype in serum, a significant number of antibody-secreting cells circulating in blood secrete IgA (34). Several studies have confirmed that IgA producing cells reactive against gut-encountered antigens can be found outside the gut (35). In the present study, the yeast diet did not have a significant effect on the level of IgA in plasma or colon content at 28 days post-weaning. However, in colon tissue, piglets fed yeast for 28 days presented a significantly lower level of IgA. This result correlates with the infiltration of neutrophils observed by histology in the same group. The ability of yeast to stimulate mucosal immunity needs further investigation, especially in long term feeding trials.

We also investigated the response of microbes and the secretion of SCFAs in pigs fed yeast using 16S rRNA gene sequencing and gas chromatography. Our results showed agreement with previous studies (36) that the microbiota of the ileum and jejunum were structurally different from that of the large intestine (colon and cecum) and the composition and metabolism of both small and large intestinal microbiota were affected by yeast diet. The availability of yeast on the intestine and the preferential substrate utilization of microbes were the major factors that affected the composition of the microbiota. In our study, we detected a difference in the alpha bacterial diversity of the large intestine between the yeast and the control groups based on the Shannon index, where the control group appeared more diverse at the amplicon level than the group fed yeast. The yeast diet markedly increased the abundance of some bacteria from the family *Lachnospiraceae, Veillonellaceae*, and *Burkholderiaceae*, while decreasing the abundance of species such as *Butyricicoccus, Fournierella, Oscillibacter, Roseburia*, and *Streptomyces* spp. in the cecum of pigs fed the yeast diet. Even though the analysis of the microbiota composition was limited to the sequencing of the 16S rRNA bacterial gene only, the overrepresentation of the *Lachnospiraceae* family might be related to the availability of the non-digested dried yeast cells in the diet.

We found that *Clostridium* was positively correlated with the high propionic levels observed in the colon of yeats fed pigs. Besides the role of *Blautia* in acetate and propionate production, our results suggest that *Dialister* and *Cupriavidus* may have a direct contribution to the propionic pool production. Propionate is used as an energy substrate of peripheral tissues, and its health effect goes beyond the gut epithelium, as it can lower serum cholesterol levels, lipogenesis, and carcinogenesis risk. Propionate may also decrease obesity (37). Some human colonic bacteria belonging to the *Negativicute*s class of Firmicutes, such as *Dialister*, have the ability to convert succinate to propionate (38). Propionate and butyrate are formed as products from rhamnose and fucose sugars or peptides and amino acid fermentation by dominant gut commensal bacteria belonging to the Lachnospiraceae, including *Roseburia* and *Blautia* species (38, 39). It is possible to speculate that the presence of yeast cell wall glucans in the feed affects the microbial diversity by selecting microbes able to degrade these glucans.

Using a KEGG pathway analysis, we found that cyanoamino and ether lipid metabolisms were significantly upregulated in the colon of pigs fed the yeast diet. Furthermore, the biosynthesis of secondary bile acids and siderophores was significantly upregulated in the cecum of pig belonging to the same group. In this regard, several studies in mice and pigs have shown that antimicrobials (AMA), besides its effect on weight gain by altering gut microbial ecology, also can cause variations in the biosynthesis of bile acids (BA) (40, 41). Accordingly, it has been suggested that alteration on BA metabolism might be the mechanism used by AMA to promote growth (41). Ipharraguerre et al. (42) have shown that the combination of zinc oxide with different AMA promotes body weight and alters the metabolism of BA, as well as improving immune tolerance and barrier function of the intestinal mucosa. These studies provide evidence that BA has an important role mediating physiological, metabolic, and immune response, hence directly affecting growth performance. Nonetheless, the effect of yeast on the biosynthesis of BA, its profile in different tissues and its impact on feed utilization or growth in piglets needs further studies, including long term feeding.

## Conclusions

The present study combining microbiome, short-chain fatty acid, and immune parameter analysis demonstrated that the diet containing high levels of yeast altered the gut microbial composition and increased the number of NK cells in the DJLN as well as the production of propionate in colon.

## Materials and Methods

### Ethical Statement

All animals were handled following the applicable laws and regulations controlling experiments with live animals in Norway (Animal Welfare Act 2009 and the local legislation derived from the directive 2010/63 EU of the European Parliament and Council of September 2010 on the protection of animals used for scientific purposes). The experiment was approved by the Norwegian Food Safety Authority (identification number 11314) and was performed at the Center for Livestock Production, Norwegian University of Life Sciences, Aas, Norway.

### Experimental Design

Forty-eight crossbred piglets (Norwegian Landrace × Yorkshire × Duroc) at ~30 days of age and an average initial body weight of 11.06 kg ± 0.84 SD, were equally distributed by litter, gender, and weight and randomly allotted to two dietary treatments, with 12 replicates per treatment. Each dietary group consisted of three replicate pens, with four piglets per pen. At the stipulated feeding times, each pig was separated from the others in an individual feeding stall for 30 min to measure individual feed intake. The experimental diets consisted of replacement of the primary sources of crude protein (CP), soybean meal, potato protein concentrate, fishmeal, and rapeseed meal with drum dried and inactivated *Cyberlindnera jadinii* corresponding to 40% of the total CP content. The yeast used in this study was produced in Lallemand, Estonia, using the lignocellulosic biomass from the Norwegian spruce tree (*Picea abies*) as a growth media as described in Cruz et al. (11). The diets were coded as control and CJ40 (40% CP from yeast). The diet composition is shown in Table S3. Piglets were fed three times per day during the first 14 days and two times per day during the remaining period. Feed was provided *ad libitum* during restrictive periods and the amounts of feed were adjusted weekly, based on the estimated feed intake of 3–5% of the live body weight. Water was accessible *ad libitum* via automatic drinkers. On the last day of the experiment, the piglets were euthanized with a captive bolt pistol. Intestinal content and tissue samples were collected for further analysis.

### Blood Sampling and Analysis

Blood samples were collected from six piglets per diet at 7 and 28 feeding days. Samples were taken in the morning 1–2 h post-prandial by venipuncture of the jugular vein while keeping the animal on its back. Both plasma and serum were collected. For serum, blood was collected using the vacutainer with gel separator (VACUETTE® TUBE CAT Serum Separator Clot Activator) and kept for 30 min in an upright position followed by centrifugation at 2,000 × g for 10 min at room temperature and immediately stored at −20°C and then later on the same day transferred to −80°C. Serum was used for the analyses of enzymes (e.g., ALT, AST) metabolites (e.g., albumin, glucose, creatinine, and urea), acute-phase protein CRP, total protein, and globulins (alpha, beta; Table S2). For plasma, blood was collected in EDTA coated vacutainer (Beckman Dickson Vacutainer System), centrifugated at 2,000 × g for 10 min at 4°C and immediately stored at −20°C and then at −80°C. Plasma samples were used for cytokine and chemokine analyses (Immunology Multiplex Assay) and IgA ELISA assay. Samples of non-centrifuged whole blood collected in EDTA vacutainer were used for hematological indices; erythrocyte count (RBC), leukocyte count (total leukocyte count and differential leukocyte count), platelets (PLT), red blood cell distribution width (RDW), hemoglobin (HGB), mean cell volume (MCV), mean corpuscular hemoglobin (MCH; Table S1).

The analysis was performed with an Advia® 2120 Hematology System using Advia 2120 MultiSpecies System Software, while serum was tested using Advia 1800 Chemistry System (Siemens healthcare diagnostics Inc., Tarrytown, NY 10591, United States) at the Central Laboratory, Norwegian University of Life Sciences.

For flow cytometry analysis, whole blood was diluted 1:1 in RPMI 1640 and kept on ice until single-cell isolation. Peripheral blood mononuclear cells (PBMCs) were recovered using a density gradient Percoll (*d* = 1.77; Sigma–Aldrich) after centrifugation at 1,200 × g, 30 min without brake at 20°C and washed twice in PBS with 2 mM EDTA. After counting, isolated PBMCs were incubated with Fixable Yellow Dead Cell Stain Kit (Life Technologies, Thermo Fisher Scientific Inc.) followed by primary monoclonal antibodies (mAbs), brief incubation with 30% normal pig serum to block Fc-receptors, and finally fluorescence-labeled secondary antibodies. To detect the intracellular CD3 epitope, surface-labeled cells were permeabilized with Intracellular Fixation and Permeabilization Buffer Set (eBioscience, Affymetrix Inc.) according to the manufacturer's instructions. Labeled cells were analyzed in a Gallios flow cytometer and data were processed using Kaluza 1.5 software (both Beckman Coulter, Inc.). Cell gates were designed to select for single and viable mononuclear cells. Defined markers were used to identify the different immune subpopulations. To detect T cells, the following antibodies were used: CD45, CD3, TCR γ/δ, CD4, CD8, FOXp3, and CD25. To identify T and NK cells, we used CD45, CD8, NKp46, CD4, Ki67, and CD27. The list of antibodies is shown in Table S4 and the gating strategy is shown in Figure S1.

Quantification of GMCSF, IFNγ, IL-1A, IL1B, IL-1RA, IL-2, IL-4, IL-6, IL-8, IL-10, IL-12, IL-18, and TNFα was measured in serum samples using MILLIPLEX MAP Porcine Cytokine and Chemokine Magnetic Bead Panel—Immunology Multiplex Assay (Merck Millipore) following the manufacturer‘s instructions.

### Gene Expression Analysis

Total RNA was extracted from colon and DJLN of twelve control diet-fed piglets and twelve yeast fed piglets following the RNeasy Plus Universal Kits protocol (Qiagen). The RNA concentration and quality were determined using NanoDrop TM 8000 spectrophotometer (Thermo Fisher Scientific) and Agilent 2100 Bioanalyzer (Agilent Technologies). All samples had a RIN-value ≥ 7. The cDNA synthesis was performed according to the AffinityScript qPCR cDNA synthesis protocol (Agilent Technologies). The qPCR reactions were performed in a total volume of 20 μL using 10 μL of LightCycler 480 SYBR Green I Master, 2 μL primers (5 μM), 3 μL H2O, and 5 μL template. The PCR conditions were as follow: 95°C 10 min, 40 cycles of 95°C 10 s, 60–64°C 10, and 72°C 10 s. As a final step, a melting curve was included. Samples were analyzed using LightCycler® 480 System (Roche Diagnostics) and calculated as 2^−ΔΔ*Cp*^. The sequences of primers used for qPCR are listed in Table S5.

### Histology

As described in detail in previously (11), at 28 days post-weaning, tissue segments were collected from the colon of 12 and 11 piglets fed either control or CJ40 diets, respectively. Formalin-fixed, paraffin-embedded tissues were cut in 4 μm thick sections before routine staining with hematoxylin and eosin. The tissue sections from the colon were evaluated histopathologically by a pathologist (CPÅ) and scored semi-quantitatively where no pathology was scored 0, very mild changes were scored 0.5; mild changes 1.0; mild-moderate changes 1.5; and moderate changes 2.0. The histopathological parameters evaluated were epithelial damage (ED); intra-epithelial lymphocytes (IEL); crypt abscess (CA); lamina propria neutrophils (LPN); lamina propria macrophages active (LPM); lamina propria lymphocytes/plasma cells (LPL); lamina propria eosinophils (LPE); and diagnosis colitis (DI).

### Short-Chain Fatty Acids (SCFAs) Analysis

The SCFAs from colon microbiota were determined by gas chromatography (Trace 1300, Thermo Fischer), equipped with a flame ionization detector. Standards of acetic acid, propionic acid, isobutyric acid, butyric acid, isovaleric acid, valeric acid, and 2-methyl valeric acid in 5% formic acid (as internal standard) were purchased from Sigma–Aldrich (Sigma–Aldrich, St. Louis, MO, United States) and were of HPLC grade with >99% purity. Before the SCFAs analysis, 500 mg of colon content were mixed with 500 μl of cold internal standard solution and sonicated for 5 min in cold water. Then, centrifugate at 4°C with 15,000 g 15 min, the supernatant was transferred to a spin column (45 kDa) centrifugate and the resulting supernatant was injected into a capillary column (30 m ×250 μm × 0.25 μm, Restek Corporation, Bellefonte, PA, USA). The column starting temperature was 90°C (2 min) followed by 10°C/min until 150°C, then 50°C/min until 250°C (1 min).

### ELISA

The concentrations of immunoglobulin A (IgA) in the plasma, colon tissue and colon content were measured with commercial kits [Pig IgA ELISA Kit (ab190536), Abcam, Cambridge, UK]. The assays were performed in duplicate and according to the manufacturer's instructions. To extract proteins from colon tissue and colon content, ~60 mg of each samples was added to a 2 mL tube containing 1 mL of lysate buffer (Tris 20 mM, NaCl 100 mM, Triton X-100 0.05%, EDTA 5 mM, and protease inhibitor cocktail) and one 5 mm Stainless Steel Beads (Qiagen, Hilden, Germany). Samples were homogenized twice for 1.5 min at 20 Hz using a TissueLyser (Qiagen, Hilden, Germany). After homogenizing, the samples were centrifuged at 4°C, 15,000 × g for 25 min. The supernatant was aliquoted into four tubes and stored at −80°C until further analyzes. Total protein concentration was measured in the supernatant from each sample using Pierce™ BCA Protein Assay Kit (Thermo Fisher Scientific, Waltham, Massachusetts, USA) following the manufacturer's instructions. Samples were normalized to the same protein concentration.

### DNA Extraction, Sequencing, and Data Processing for Microbiota Profiling

At the end of the experiment, digesta of ileum, jejunum, colon, and cecum, were collected and immediately frozen in liquid nitrogen and then stored at −80°C until processing. Total genomic DNA was extracted using QIAamp Fast DNA Stool kit (QIAGEN) according to the manufacturer's instructions. The extracted DNA was quantified, accessed for purity and used for 16S metabarcoding with the primers specific to V1-V3 hypervariable region of 16S rRNA gene (27F−5′-AGAGTTTGATCCTGGCTCAG−3′ and 534R−5′-ATTACCGCGGCTGCTGG3′). The amplicons were generated using Illumina 2 × 300 bp chemistry at GATC-Biotech, Germany, but only forward reads were used for subsequent data analysis.

The analysis of microbial communities was done using 2018.8 version of the Quantitative Insights into Microbial Ecology (QIIME2) pipeline (43). The taxonomic analysis was performed using 99% full-length sequences of the GreenGenes 13.8 bacterial 16S subset database (44). The samples from the ileum, jejunum, colon, and cecum were analyzed separately. Raw sequence reads were quality score filtered to retain sequences with quality score > Q20. The sequence quality control and OTU table construction were performed using DADA2 (45) algorithm within QIIME2 pipeline. The resulting OTU tables were used for subsequent α and β diversity analysis. For differential abundance analysis, the OTU tables were filtered to retain sequences occurring in at least three samples and having at least 11 copies per dataset. The filtered OTU tables were further used to estimate α and β diversity in the filtered datasets to ensure that the filtering of the datasets did not affect initial biological conclusions. The filtered datasets were then used for differential abundance analysis with Gneiss algorithm (46). The relative frequencies of differentially abundant taxa and concentrations of propionic acid measured in colon samples were used for correlation analysis through the calculation of the Pearson correlation coefficient (47) and respective *p*-values (48). The calculation of correlation metrics and visualization were performed in R using ggplot2 package (49). Only correlation coefficients with *p* ≤ 0.05 were used to draw conclusions.

The differentially abundant taxa were manually curated and analyzed for their potential functions using KEGG database (50) integrated into PICRUSt 1.1.3 pipeline (51). Predicted functions were hierarchically collapsed into KEGG level 3 pathways using standard PICRUSt tools and further analyzed using the two-tailed homoscedastic *t*-test in R. The raw sequencing data were deposited at NCBI nucleic acid collection under the bioproject PRJNA531397.

### Statistical Analysis

Non-parametric data from flow cytometry were analyzed by Kruskal–Wallis followed by *post-hoc* Dunn's test with a comparison of mean rank. D'Agostino and Pearson normality test was used to test the normal distribution of data from the histopathological scoring. A non-parametric Mann–Whitney test was used to compare the mean scores of the histopathological parameters. The qPCR results were analyzed using Student *t*-test, were *p* ≤ 0.05 was considered a significant difference.

## Data Availability Statement

The original contributions presented in the study are publicly available. This data can be found here: https://www.ncbi.nlm.nih.gov/, PRJNA531397.

## Ethics Statement

The animal study was reviewed and approved by the Norwegian Food Safety Authority (identification number 11314).

## Author Contributions

LL, AS, AB, CP, and MØ designed the experiment. LL, AS, and AB conducted animal study, lab, and statistical analysis. LL, AS, AB, CÅ, CP, and RÅ participate in the sampling and analysis. AB performed sequencing and bioinformatic analysis. LL and AS data visualization. LL and AB wrote the manuscript with the input of other co-authors. All the authors read and approved the final manuscript.

## Conflict of Interest

The authors declare that the research was conducted in the absence of any commercial or financial relationships that could be construed as a potential conflict of interest.
