# Supplementary figures and images for: Cyberlindnera jadinii Yeast as a Protein Source for Weaned Piglets—Impact on Immune Response and Gut Microbiota
Source: Front Immunol. 2020 Sep 2;11:1924. doi: 10.3389/fimmu.2020.01924 (PMC7495143; doi:10.3389/fimmu.2020.01924)

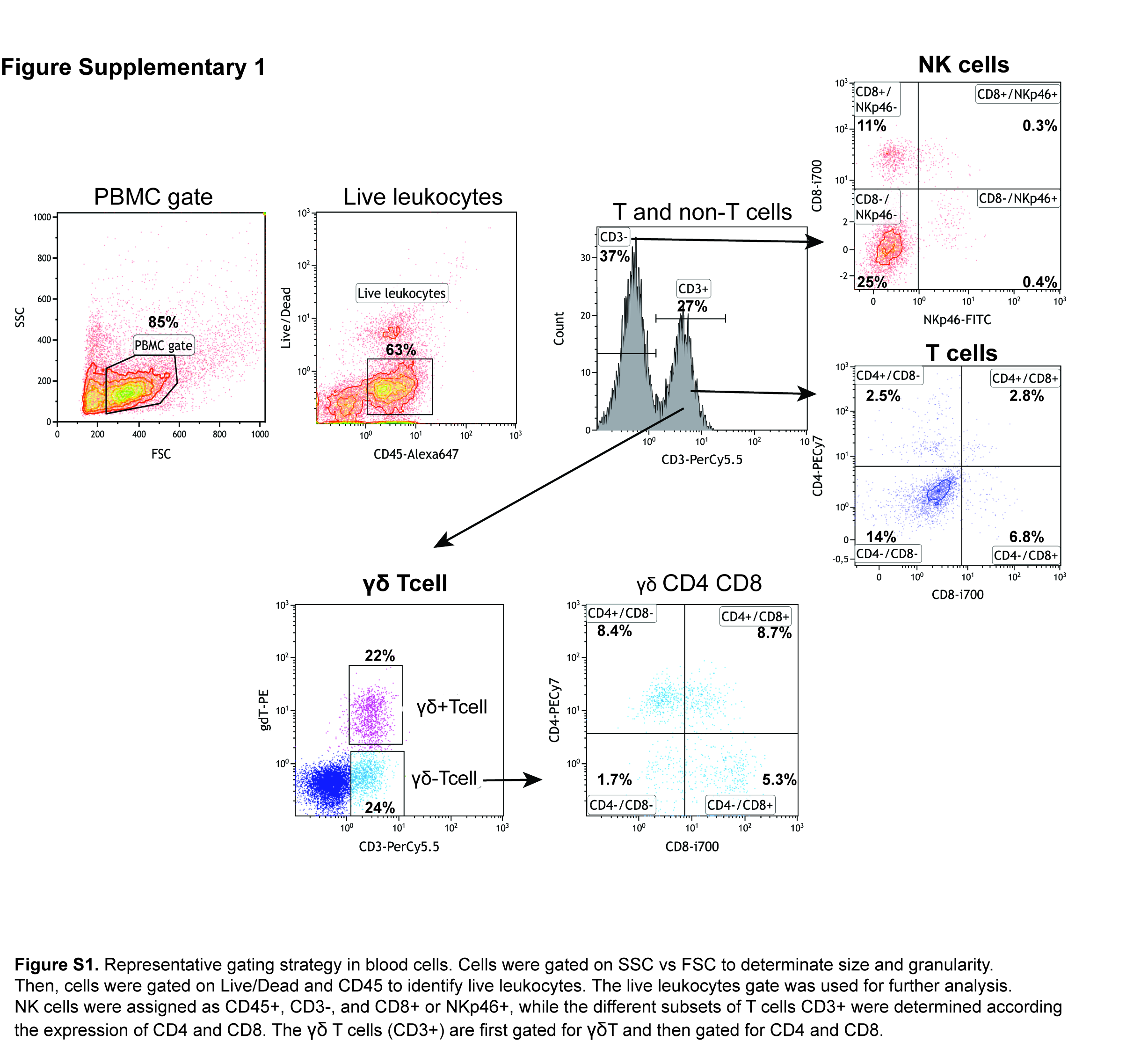

Supplement: Supplementary file 1 [file Image_1.TIF]

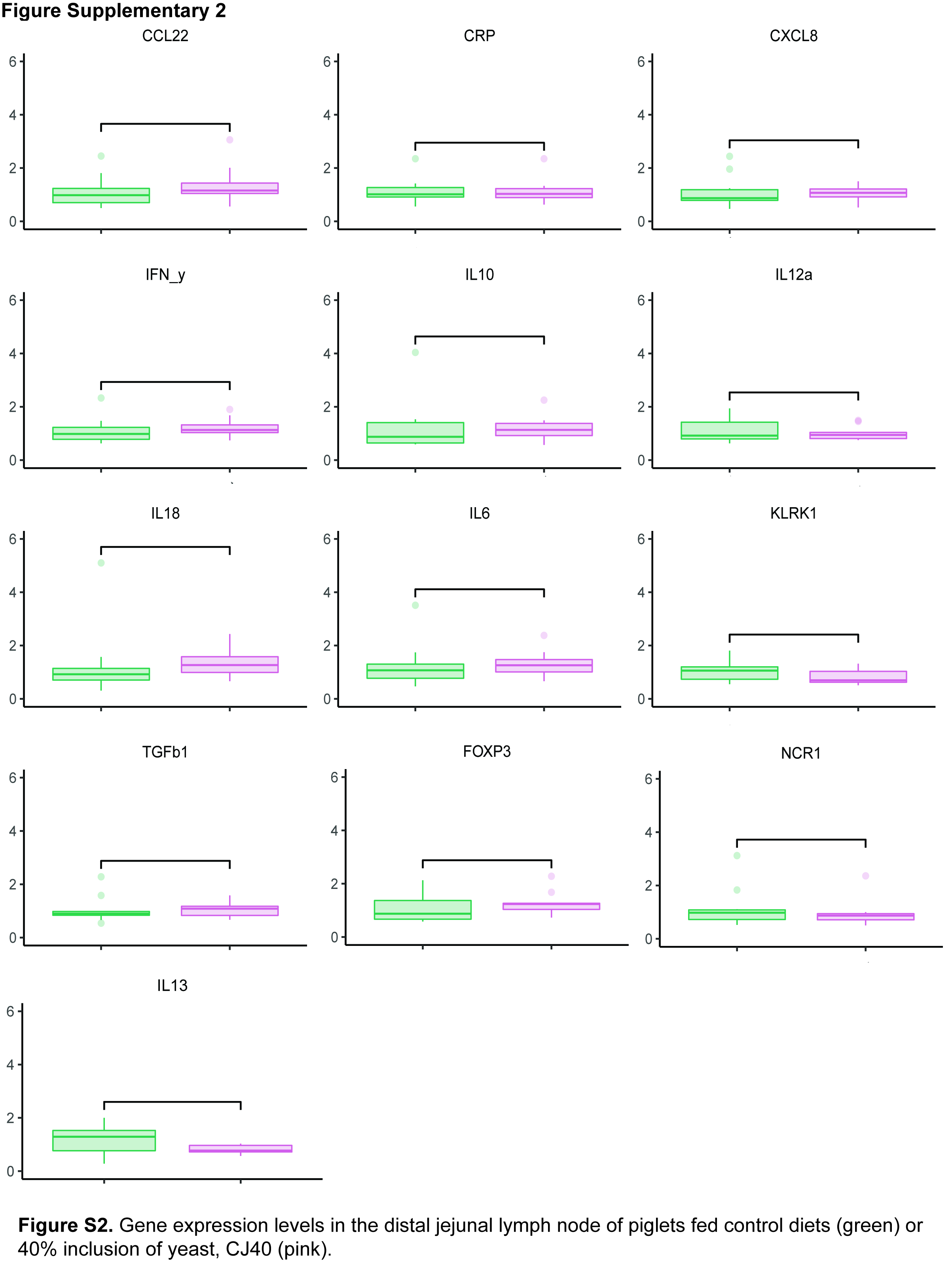

Supplement: Supplementary file 2 [file Image_2.TIF]

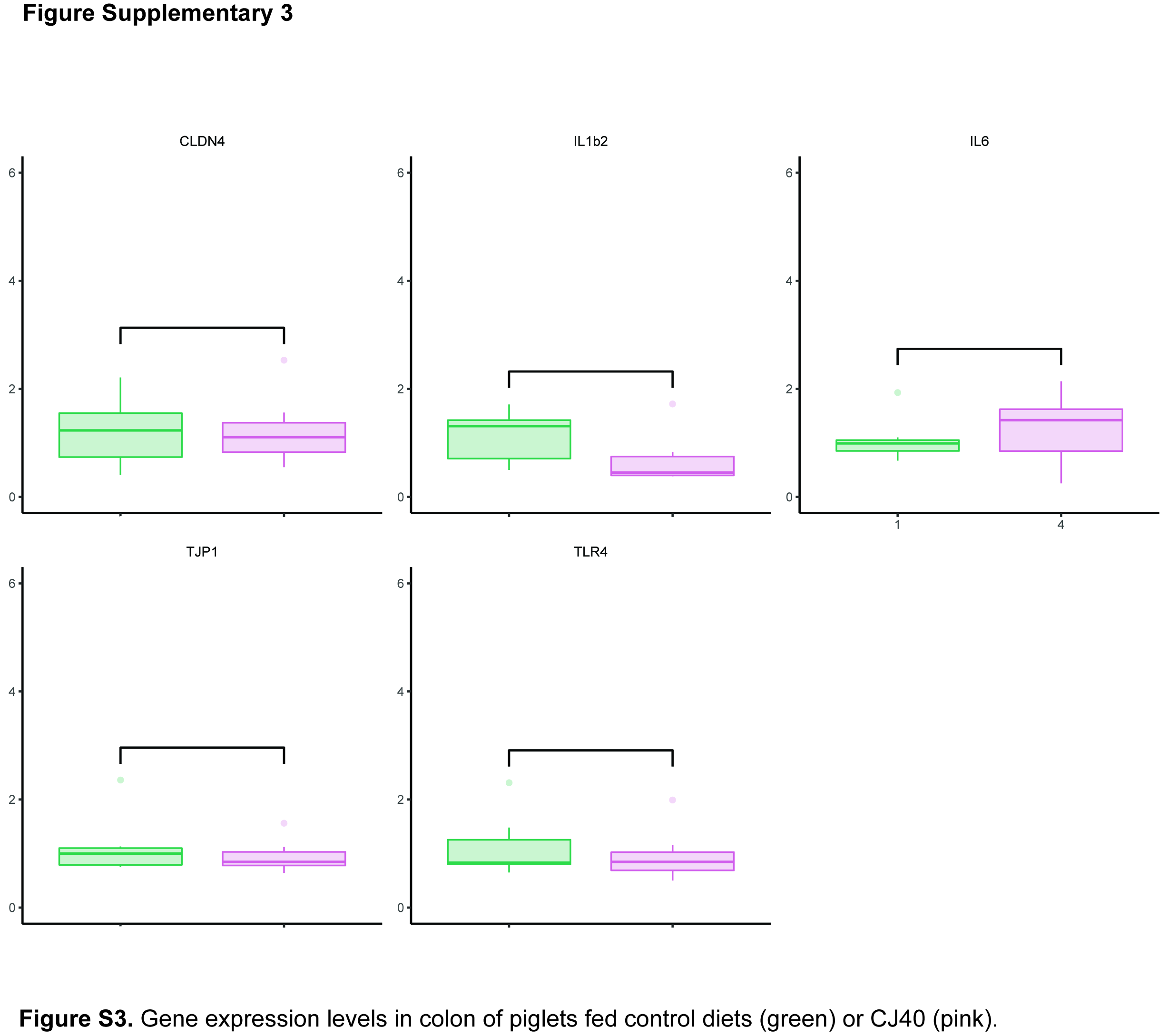

Supplement: Supplementary file 3 [file Image_3.TIF]
