# Supplementary material for: Cyberlindnera jadinii Yeast as a Protein Source for Weaned Piglets—Impact on Immune Response and Gut Microbiota
Source: Front Immunol. 2020 Sep 2;11:1924. doi: 10.3389/fimmu.2020.01924 (PMC7495143; doi:10.3389/fimmu.2020.01924)
Supplement: Supplementary file 4 [file Data_Sheet_1.docx]

| **day** | **Pig ID** | **Diet** | **WBC** | **RBC** | **HGB** | **HCT** | **MCV** | **MCHC** | **RDW** | **PLT** | **#Neut** | **#Lymf** | **#Mono** | **#Eos** | **#Baso** | **#LUC** |
| --- | --- | --- | --- | --- | --- | --- | --- | --- | --- | --- | --- | --- | --- | --- | --- | --- |
|  |  |  | **x 10e9** | **x 10e1** | **g/L** | **L/L** | **fL** | **g/L** | **%** | **x 10e9** | **x 10e9** | **x 10e9** | **x 10e9** | **x 10e9** | **x 10e9** | **x 10e9** |
| 7 | 592 | 1 | 11.3 | 7.00 | 135 | 0.38 | 54.4 | 356 | 17.5 | 466 | 5.0 | 5.7 | 0.4 | 0.1 | 0.1 | 0.1 |
| 7 | 617 | 1 | 14.5 | 6.97 | 127 | 0.37 | 53.2 | 343 | 24.7 | 417 | 6.2 | 7.6 | 0.4 | 0.1 | 0.1 | 0.2 |
| 7 | 625 | 1 | 17.3 | 8.80 | 107 | 0.35 | 40.0 | 304 | 29.7 | 580 | 8.7 | 7.6 | 0.6 | 0.0 | 0.1 | 0.3 |
| 7 | 658 | 1 | 12.0 | 6.94 | 125 | 0.36 | 52.2 | 344 | 19.4 | 322 | 3.9 | 7.2 | 0.5 | 0.2 | 0.1 | 0.1 |
| 7 | 712 | 1 | 27.8 | 7.37 | 134 | 0.41 | 55.8 | 326 | 18.5 | 617 | 8.8 | 17.4 | 0.8 | 0.2 | 0.3 | 0.4 |
| 7 | 733 | 1 | 13.8 | 7.61 | 138 | 0.41 | 54.2 | 335 | 17.8 | 451 | 6.0 | 6.4 | 1.0 | 0.1 | 0.2 | 0.1 |
| **Mean** |  |  | 16.1 | 7.4 | 127.7 | 0.4 | 51.6 | 334.7 | 21.3 | 475.5 | 6.4 | 8.7 | 0.6 | 0.1 | 0.2 | 0.2 |
| **SD** |  |  | 2.3 | 0.3 | 4.2 | 0.009 | 2.2 | 6.7 | 1.8 | 40.3 | 0.7 | 1.6 | 0.09 | 0.03 | 0.03 | 0.05 |
| 7 | 583 | CJ40 | 22.4 | 7.83 | 145 | 0.44 | 56.1 | 330 | 21.4 | 384 | 6.8 | 13.8 | 1.0 | 0.2 | 0.5 | 0.1 |
| 7 | 618 | CJ40 | 15.1 | 7.48 | 121 | 0.37 | 48.9 | 332 | 24.9 | 437 | 5.7 | 8.4 | 0.6 | 0.1 | 0.1 | 0.2 |
| 7 | 634 | CJ40 | 14.7 | 6.93 | 124 | 0.37 | 52.9 | 337 | 20.1 | 526 | 6.4 | 7.3 | 0.6 | 0.1 | 0.2 | 0.2 |
| 7 | 713 | CJ40 | 15.6 | 6.50 | 118 | 0.35 | 53.4 | 339 | 20.8 | 660 | 5.3 | 9.4 | 0.6 | 0.1 | 0.1 | 0.1 |
| 7 | 721 | CJ40 | 15.6 | 7.02 | 125 | 0.37 | 52.1 | 340 | 18.7 | 287 | 8.0 | 6.6 | 0.6 | 0.2 | 0.1 | 0.1 |
| 7 | 738 | CJ40 | 16.6 | 8.12 | 136 | 0.43 | 52.4 | 318 | 24.7 | 418 | 6.4 | 9.0 | 0.8 | 0.2 | 0.2 | 0.1 |
| **Mean** |  |  | 16.7 | 7.3 | 128.2 | 0.4 | 52.6 | 332.7 | 21.8 | 452 | 6.4 | 9.1 | 0.7 | 0.2 | 0.2 | 0.1 |
| **SD** |  |  | 1.07 | 0.23 | 3.8 | 0.01 | 0.86 | 3.05 | 0.94 | 47.7 | 0.35 | 0.94 | 0.06 | 0.02 | 0.05 | 0.02 |
| 28 | 592 | 1 | 14.1 | 6.33 | 115 | 0.33 | 52.0 | 348 | 17.6 | 531 | 5.3 | 8.1 | 0.4 | 0.1 | 0.1 | 0.2 |
| 28 | 617 | 1 | **Clog** |  |  |  |  |  |  |  |  |  |  |  |  |  |
| 28 | 733 | 1 | 18.9 | 7.52 | 129 | 0.38 | 50.8 | 338 | 17.4 | 395 | 4.1 | 13.1 | 1.1 | 0.2 | 0.3 | 0.1 |
| 28 | 625 | 1 | 14.3 | 9.18 | 127 | 0.39 | 42.9 | 324 | 26.3 | 437 | 5.5 | 7.8 | 0.7 | 0.1 | 0.1 | 0.1 |
| 28 | 658 | 1 | 15.0 | 6.98 | 118 | 0.35 | 50.7 | 333 | 18.4 | 510 | 4.5 | 9.2 | 0.9 | 0.2 | 0.2 | 0.1 |
| 28 | 712 | 1 | 33.2 | 7.23 | 127 | 0.38 | 52.3 | 336 | 17.8 | 606 | 16.6 | 15.2 | 0.5 | 0.4 | 0.2 | 0.4 |
| **Mean** |  |  | 19.1 | 7.4 | 123.2 | 0.4 | 49.7 | 335.8 | 19.5 | 495.8 | 7.2 | 10.7 | 0.7 | 0.2 | 0.2 | 0.2 |
| **SD** |  |  | 3.25 | 0.42 | 2.5 | 0.01 | 1.55 | 3.5 | 1.5 | 32.9 | 2.11 | 1.31 | 0.11 | 0.04 | 0.03 | 0.05 |
| 28 | 583 | CJ40 | 15.2 | 6.74 | 113 | 0.34 | 49.8 | 338 | 20.4 | 413 | 3.6 | 10.5 | 0.8 | 0.1 | 0.2 | 0.1 |
| 28 | 618 | CJ40 | 19.1 | 7.97 | 126 | 0.37 | 47.0 | 336 | 21.8 | 419 | 5.9 | 12.1 | 0.7 | 0.2 | 0.2 | 0.1 |
| 28 | 634 | CJ40 | 20.6 | 7.43 | 125 | 0.38 | 50.6 | 332 | 18.2 | 415 | 3.4 | 15.2 | 1.3 | 0.1 | 0.3 | 0.1 |
| 28 | 713 | CJ40 | 24.2 | 7.10 | 126 | 0.37 | 52.3 | 338 | 19.7 | 382 | 5.3 | 17.1 | 1.2 | 0.2 | 0.3 | 0.1 |
| 28 | 721 | CJ40 | 24.9 | 6.55 | 107 | 0.31 | 47.7 | 343 | 18.9 | 316 | 13.7 | 9.8 | 0.6 | 0.2 | 0.3 | 0.3 |
| 28 | 738 | CJ40 | 14.8 | 7.39 | 120 | 0.37 | 50.1 | 325 | 20.0 | 394 | 5.7 | 8.1 | 0.5 | 0.2 | 0.1 | 0.3 |
| **Mean** |  |  | 19.8 | 7.2 | 119.5 | 0.4 | 49.6 | 335.3 | 19.8 | 389.8 | 6.3 | 12.1 | 0.9 | 0.2 | 0.2 | 0.2 |
| **SD** |  |  | 1.6 | 0.19 | 2.95 | 0.009 | 0.72 | 2.3 | 0.46 | 14.48 | 1.41 | 1.27 | 0.12 | 0.01 | 0.03 | 0.03 |

**Table S1.** Hematology parameters of pig fed control diet (1) or 40% inclusion of yeast (CJ40).

| **day** | **Pig ID** | **Diet** | **AST** | **AP** | **GGT** | **GD** | **CK** | **Urea** | **Krt** | **Tbil** | **Glu** | **Kol** | **uorgP** | **Ca** | **CRP** | **T**  **prt** | **Alb -** | **Alph** | **Alph** | **Beta 2** | **Gamm** |
| --- | --- | --- | --- | --- | --- | --- | --- | --- | --- | --- | --- | --- | --- | --- | --- | --- | --- | --- | --- | --- | --- |
|  |  |  | U/L | U/L | U/L | U/L | U/L | mmol/L | µmol/L | µmol/L | mmol/L | mmol/L | mmol/L | mmol/L | mg/L | g/L | g/L | g/L | g/L | g/L | g/L |
| 7 | 583 | CJ40 | 31 | 407 | 40 | 2 | 486 | 2.2 | 103 | 0 | 8.2 | 2.3 | 3 | 2.8 | 32.2 | 51 | 29.2 | 3.7 | 6 | 5.2 | 3.3 |
| 7 | 618 | CJ40 | 22 | 314 | 38 | 2 | 354 | 1.1 | 101 | 1 | 7.7 | 2.1 | 3.4 | 2.7 | 20.7 | 50 | 27.3 | 3.4 | 6.4 | 5.9 | 4.3 |
| 7 | 634 | CJ40 | 31 | 399 | 39 | 1 | 281 | 2.4 | 98 | 0 | 6.4 | 2.1 | 3.3 | 2.7 | 3.4 | 46 | 26.6 | 2.6 | 5.5 | 4.9 | 3.7 |
| 7 | 713 | CJ40 | 26 | 306 | 28 | 2 | 323 | 0.9 | 102 | 0 | 6.9 | 2.3 | 3.3 | 2.5 | 1.1 | 48 | 26 | 3.1 | 5.7 | 6 | 4.8 |
| 7 | 721 | CJ40 | 30 | 216 | 31 | 1 | 490 | 2.3 | 99 | 1 | 7 | 2 | 2.4 | 2.6 | 31.8 | 46 | 24.3 | 3.2 | 6.9 | 5.3 | 3 |
| 7 | 738 | CJ40 | 50 | 434 | 28 | 2 | 331 | 4.2 | 117 | 1 | 8.7 | 2 | 3.1 | 2.6 | 40.9 | 51 | 30.2 | 3 | 5.5 | 6.9 | 2.9 |
| **Mean** |  |  | 32 | 346 | 34 | 2 | 378 | 2 | 103 | 1 | 7 | 2 | 3 | 3 | 22 | 49 | 27 | 3.2 | 6 | 6 | 4 |
| **SD** |  |  | 3.6 | 30.6 | 2. | 0.2 | 33 | 0.4 | 2.5 | 0.2 | 0.3 | 0.05 | 0.13 | 0.03 | 6.1 | 0.8 | 0.8 | 0.1 | 0.2 | 0.3 | 0.3 |
| 7 | 592 | 1 | 31 | 303 | 40 | 1 | 361 | 3.8 | 109 | 1 | 6.9 | 1.6 | 2.9 | 2.6 | 6.7 | 48 | 26.1 | 3.6 | 6.5 | 5.3 | 3.4 |
| 7 | 617 | 1 | 21 | 214 | 22 | 2 | 174 | 3.3 | 110 | 2 | 6.8 | 1.8 | 2.7 | 2.7 | 21.1 | 53 | 29.2 | 4.2 | 5.9 | 6.9 | 3.5 |
| 7 | 625 | 1 | 29 | 450 | 42 | 1 | 338 | 2.5 | 102 | 0 | 8.9 | 2.4 | 3.1 | 2.9 | 21 | 57 | 30.3 | 3.7 | 7.6 | 8.7 | 4.1 |
| 7 | 658 | 1 | 25 | 429 | 34 | 3 | 228 | 1.2 | 106 | 1 | 7.3 | 1.8 | 3.2 | 2.7 | 15.7 | 51 | 26.4 | 3.3 | 5.5 | 6.3 | 6.2 |
| 7 | 712 | 1 | 27 | 321 | 40 | 1 | 286 | 1.2 | 128 | 0 | 6.1 | 2.3 | 3.3 | 2.7 | 3.6 | 52 | 28.6 | 2.9 | 7.4 | 5.5 | 4.5 |
| 7 | 733 | 1 | 52 | 257 | 26 | 2 | ### | 2.9 | 109 | 0 | 6.4 | 2.1 | 2.4 | 2.7 | 21.4 | 55 | 30.1 | 4.4 | 6.2 | 6.8 | 3.6 |
| **Mean** |  |  | 31 | 329 | 34 | 2 | 277 | 2 | 111 | 1 | 7 | 2 | 3 | 3 | 15 | 53 | 28 | 4 | 7 | 7 | 4 |
| **SD** |  |  | 4 | 34.9 | 3.09 | 0.3 | 30.9 | 0.4 | 3.3 | 0.3 | 0.3 | 0.1 | 0.1 | 0.03 | 2.9 | 1.1 | 0.6 | 0.2 | 0.3 | 0.4 | 0.3 |
| 28 | 583 | CJ40 | 36 | 296 | 37 | 2 | 606 | 1.3 | 78 | 0 | 8.7 | 2.6 | 3.8 | 2.9 | 28.9 | 51 | 27.5 | 3.8 | 7 | 5.8 | 2.9 |
| 28 | 618 | CJ40 | 27 | 257 | 44 | 2 | 765 | 0.8 | 94 | 0 | 11.5 | 2.5 | 3.7 | 2.6 | 25 | 49 | 25 | 3.8 | 7.2 | 5.9 | 4 |
| 28 | 634 | CJ40 | 35 | 335 | 42 | 2 | 359 | 1 | 94 | 0 | 12.4 | 2.5 | 3.8 | 2.7 | 44.4 | 47 | 24.4 | 3.3 | 6.3 | 6 | 3.4 |
| 28 | 713 | CJ40 | 68 | 278 | 36 | 2 | ### | 0.9 | 48 | 0 | 9.8 | 2.9 | 3.9 | 2.7 | 8 | 51 | 26 | 3.1 | 6.8 | 6.9 | 4 |
| 28 | 721 | CJ40 | 36 | 250 | 38 | 2 | 646 | 2.1 | 79 | 0 | 7.1 | 2.3 | 3.9 | 2.6 | 39.8 | 46 | 22.7 | 3.5 | 7.1 | 5.8 | 3.6 |
| 28 | 738 | CJ40 | 40 | 463 | 35 | 1 | 261 | 4.8 | 88 | 0 | 7 | 1.8 | 3.4 | 2.7 | 49.3 | 45 | 24.7 | 2.8 | 5.2 | 5.9 | 3.5 |
| **Mean** |  |  | 40 | 313 | 39 | 2 | 527 | 2 | 80 | 0 | 9 | 2 | 4 | 3 | 33 | 48 | 25 | 3 | 7 | 6 | 4 |
| **SD** |  |  | 5.2 | 29.6 | 1.3 | 0.1 | 83.9 | 0.5 | 6.4 | 0 | 0.8 | 0.1 | 0.06 | 0.04 | 5.6 | 0.9 | 0.6 | 0.1 | 0.3 | 0.1 | 0.1 |
| 28 | 592 | 1 | 44 | 229 | 38 | 2 | 499 | 1.3 | 81 | 0 | 8.1 | 2.4 | 4 | 2.7 | 23.7 | 48 | 24.1 | 3.7 | 6.5 | 6.2 | 4.1 |
| 28 | 617 | 1 | 70 | 209 | 23 | 2 | ### | 1 | 78 | 0 | 10.5 | 2.8 | 3.6 | 2.8 | 44.1 | 49 | 24.7 | 3.4 | 6.5 | 7.4 | 4 |
| 28 | 625 | 1 | 30 | 321 | 43 | 1 | 315 | 1.8 | 95 | 0 | 6.9 | 2.9 | 3.9 | 2.9 | 27.9 | 53 | 27.9 | 3.7 | 7.7 | 7.3 | 3.6 |
| 28 | 658 | 1 | 34 | 264 | 39 | 2 | 820 | 0.8 | 79 | 0 | 8 | 2.6 | 3.6 | 2.8 | 18.8 | 51 | 25.6 | 4 | 5.7 | 6.5 | 5 |
| 28 | 712 | 1 | 28 | 247 | 44 | 1 | 392 | 1.2 | 84 | 0 | 6.2 | 2.1 | 4.1 | 2.8 | 130.7 | 53 | 23.1 | 5.7 | 8.3 | 7 | 4.7 |
| 28 | 733 | 1 | 42 | 324 | 37 | 2 | 912 | 1.1 | 87 | 0 | 8.5 | 3 | 3.7 | 2.8 | 29.5 | 54 | 28.6 | 3 | 7.2 | 6.5 | 4.4 |
| **Mean** |  |  | 41 | 266 | 37 | 2 | 588 | 1 | 84 | 0 | 8 | 3 | 4 | 3 | 46 | 51 | 26 | 4 | 7 | 4 | 4 |
| **SD** |  |  | 5.7 | 17.7 | 2.8 | 0.2 | 105 | 0.1 | 2.3 | 0 | 0.5 | 0.1 | 0.07 | 0.1 | 15.8 | 0.9 | 0.8 | 0.3 | 0.3 | 0.2 | 0.1 |

**Table 2S.** Biochemical parameters of blood at 7 and 28 days postweaning. Diet 1: Control diet; CJ40: Diet supplemented with 40% yeast.

**Table S3.** Dietary composition of the experimental diets.

| **Item** | Control | CJ40 |
| --- | --- | --- |
| **Ingredient, g/kg as fed basis** |  |  |
| Wheat | 624 | 593 |
| Barley | 100 | 100 |
| Oats | 50 | 50 |
| Soybean meal^1^ | 80 | 19 |
| Potato protein conc.^2^ | 38 | 9 |
| Fish meal^3^ | 20 | 5 |
| Rapeseed meal^4^ | 20 | 5 |
| Yeast - *Cyberlindnera jadinii ^5^* | ⎯ | 146 |
| Rapeseed oil | 22 | 25 |
| Monocalcium phosphate | 13 | 16 |
| Limestone | 9 | 9 |
| Sodium chloride | 6 | 5 |
| Iron (Fe) | 0.4 | 0.4 |
| Vitamin+mineral premix^6^ | 4.8 | 5.0 |
| L-Lysine | 6.3 | 5.8 |
| L-Methionine | 2.1 | 3.0 |
| L-Threonine | 2.8 | 2.4 |
| L-Valine | 1.0 | 1.0 |
| L-Tryptophan | 0.9 | 1.0 |
| *Calculated content* |  |  |
| Net energy (MJ/kg) | 9.94 | 9.94 |
| Crude protein | 170 | 170 |
| Crude protein from yeast (%) | 0.00 | 40.3 |

**^1^** Non-GMO soybean meal, Denofa AS, Fredrikstad, Norway: DM 881 g/kg, CP 458 g/kg, crude fat 10 g/kg, ash 56 g/kg, neutral detergent fiber (NDF) 89 g/kg, gross energy 17.5 MJ/kg. **^2^** Cargill, Denmark: DM 914 g/kg, CP 725 g/kg, crude fat 30 g/kg, ash 20 g/kg, gross energy 21.8 MJ/kg. **^3^** Norsildmel AS, Egersund, Norway: DM 917 g/kg, CP 684 g/kg, crude fat 73 g/kg, ash 145 g/kg, NDF 5 g/kg, gross energy 19.4 MJ/kg. **^4^** Expeller pressed rapeseed meal, Mestilla, UAB, Klaipeda, Lithuania: DM 889 g/kg, CP 350 g/kg, crude fat 88 g/kg, ash 59 g/kg, NDF 161 g/kg, gross energy 19.1 MJ/kg. **^5^** Dried inactivated *Cyberlindnera jadinii*: dry matter (DM) 970 g/kg, CP (N×6.25) 470 g/kg, crude fat 16 g/kg, ash 78 g/kg, gross energy 19.9 MJ/kg; essential amino acid content in g/16 g N: 24.4 Arg, 8.5 His, 21.6 Ile, 31.6 Leu, 30.6 Lys, 5.2 Met, 18.4 Phe, 25.6 Thr, 25.9 Val, 6.2 Trp. **^6^** Provided per kg of diet: 120 mg Fe (FeSO4); 60 mg Mn (MnO); 120 mg Zn (ZnO); 26 mg Cu (CuSO4); 0.60 mg I (Ca (IO3)); <1.0 g Se; 8000 IU vitamin A; 45 mg dl-α-tocopheryl acetate; 105 mg ascorbic acid; 1500 IU cholecalciferol; 4.64 mg menadione; 3 mg thiamin; 5.63 mg riboflavin; 45 mg niacin; 15 mg pantothenic acid; 20 μg cyanocobalamin.

| **Antigen** | **Clone** | **Isotype** | **Fluorochrome** | **Labeling strategy** | **Source of primary Ab** |
| --- | --- | --- | --- | --- | --- |
| CD14 | Tük4 | mIgG2a | VioBlue | Directly conjugated | Miltenyi Biotec |
| CD163 | 2A10/11 | mIgG1 | FITC | Directly conjugated | LSBio |
| CD45 | K252.1E4 | IgG1 | Alexafluor647 | Directly conjugated | Bio-Rad |
| CD3 | BB23-8E6-8C8 | mIgG2a | PerCp-Cy5.5 | Directly conjugated | BD Biosciences |
| SLA-DR (MHCII) | 2E9/13 | mIgG2b | PE | Secondary Ab | Bio-Rad |
| gdT-cells | MAC320 | rIgG2a | PE | Directly conjugated | BD Biosciences |
| CD4a | 74-12-4 | IgG2b | PE-Cy7 | Directly conjugated | BD Biosciences |
| CD8a | 76-2-11 | mIgG2a | i700 | Biotin-streptavidin | SouthernBiotech |
| Foxp3 | FJK-16S | mIgG2a | eFluor450 | Directly conjugated | ThermoFisher Scientific |
| CD25 | P4A10 | IgG1 | FITC | Directly conjugated | eBioscience |
| NKp46 | VIV-KM1 | mIgG1 | FITC | Directly conjugated | Bio-Rad |
| Ki67 | MKI67 | IgG1 | V450 | Directly conjugated | BD Biosciences |
| CD27 | B30C7 | MIgG1 | RPE | Directly conjugated | Bio-Rad |

**Table S4.** Primary antibodies and secondary reagents used for flow cytometry analyses.

**Table S5.** List of primers used for qPCR analysis.

| Gene | Sequence (5’ → 3’ | Product size (bp) | GenBank accession number |
| --- | --- | --- | --- |
| CLDN4 | F: GCCCTCATCGTCATCTGTATC | 92 | XM_005661969.2 |
|  | R:CTCTCATCATCCACGCAGTT |  |  |
| TLR4 | F:TTTCCACAAAAGTCGGAAGG | 145 | NM_001293316.1 |
|  | R:CAACTTCTGCAGGACGATGA |  |  |
| TJP1 | F:CGGAAAAGTGCCAGGAAAT | 118 | XM_021098896.1 |
|  | R:TCAATGCTCCATACCAACCA |  |  |
| IL-8(CXCL8) | F:GCTCTCTGTGAGGCTGCAGTTC | 79 | NM_213867.1 |
|  | R:AAGGTGTGGAATGCGTATTTATGC |  |  |
| NCR1 | F:GAGGCAAAAACCAGCCAAT | 166 | XM_005664831.3 |
|  | R:AGACCCAAAGAATAAGGCAAAGT |  |  |
| CRP | F:GGTGGGAGACATTGGAGATG | 85 | NM_213844.2 |
|  | R:GAAGGTCCCACCAGCATAGA |  |  |
| IFN-y | F: CCATTCAAAGGAGCATGGAT | 146 | NM_213948.1 |
|  | R: GAGTTCACTGATGGCTTTGC |  |  |
| IL1b2 | F:ACCCTGCAGCTGGAGGAT | 182 | NM_001302388.2 |
|  | R:CCTTTGGAGTTTCCCAGGA |  |  |
| IL6 | F:CTGCTTCTGGTGATGGCTACT | 144 | NM_001252429.1 |
|  | R:AGAGATTTTGCCGAGGATGT |  |  |
| IL10 | F:CAGAGAGTGATGGGGAGGAT | 122 | NM_214041.1 |
|  | R:GGCCTTGCTCTTGTTTTCAC |  |  |
| IL13 | F:TCACCCAGAACCAGAAGACA | 113 | XM_005661643.3 |
|  | R:GCAGTCGGAGATGTTGATGA |  |  |
| IL18 | F:GAAATCTGAACGACCAAGTCCTTT | 101 | XM_005667327.2 |
|  | R:TACGGTCTGAGGTGCATTATCTGA |  |  |
| CCL22 | F:CCTGCGTGTGGTGAAGTATCA | 120 | NM_001256776.2 |
|  | R:TTCTTCACCCAGGGCAGTCT |  |  |
| FOXP3 | F:GGTGCAGTCTCTGGAACAAC | 148 | XM_021079539.1 |
|  | R:GGTGCCAGTGGCTACAATAC |  |  |
| TGFB1 | F:AGGTCACCCGCGTGCTAAT | 101 | XM_021093503.1 |
|  | R:GAGCTCCGACGTGTTGAACAG |  |  |
| KLRK1 | F:ACAGCAGAGAAGACCAGGATTTCTTCA | 104 | XM_021091111.1 |
|  | R:GGAACCATCTTCCCACTGCCAGG |  |  |
| NCR2 | F:TCCGTGAGGTTCCATCTGGCCGTGT | 140 | XM_021098670.1 |
|  | R:TGTGAAAGGGCAGCGATGGCAGAGG |  |  |
| IL2 | F:TCAACTCCTGCCACAATGT | 89 | XM_021100436.1 |
|  | R:CTTGAAGTAGGTGCACCGT |  |  |
| IL12a | F:AGCCACGAATGAGAGTTGC | 107 | NM_213993.1 |
|  | R:TGCTAAGGCACAGGGTTGT |  |  |
| GAPDH | F:ACACTCACTCTTCTACCTTTG | 90 | XM_021091114.1 |
|  | R:CAAATTCATTGTCGTACCAG |  |  |
| OCLN | F: CTGCCTTCTGCTTCATTGCT | 127 | XM_005672525.3 |
|  | R: AACACCATCACACCCAGGAT |  |  |
